# Initial evaluation of an intervention to address provider implicit bias in pediatric sickle cell disease pain care: A mixed methods pilot study

**DOI:** 10.1080/24740527.2025.2486819

**Published:** 2025-05-09

**Authors:** Siddika S. Mulchan, Christopher B. Theriault, Susan DiVietro, Mark D. Litt, Emily O. Wakefield, Javeed Sukhera, Paula Tanabe, Hannah R. Thomas, Melissa Santos, William T. Zempsky, Donna Boruchov, Adam T. Hirsh

**Affiliations:** a Hematology/Oncology, Connecticut Children’s Medical Center, Hartford, Connecticut, USA; b Department of Pediatrics, University of Connecticut School of Medicine, Farmington, Connecticut, USA; c Injury Prevention Center, University of Connecticut, Storrs, Connecticut, USA; d Department of Behavioral Sciences, UConn Health, Farmington, Connecticut, USA; e Department of Psychiatry, Hartford Hospital, Hartford, Connecticut, USA; f Department of Medicine, Duke University School of Nursing and Medicine, Durham, North Carolina, USA; g Department of Psychological Sciences, University of Connecticut, Storrs, Connecticut, USA; h Psychology, Indiana University Indianapolis, Indianapolis, Indiana, USA

**Keywords:** Sickle cell disease, pediatrics, implicit bias, mixed methods

## Abstract

**Background:**

Health care provider (HCP) implicit bias can impact health outcomes for youth with sickle cell disease (SCD).

**Aims:**

: The aim of this study was to evaluate the feasibility, acceptability, and preliminary impact of an individuation and perspective-taking (IPT) intervention to decrease implicit bias and improve pain treatment clinical decision making in pediatric SCD HCPs.

**Methods:**

This mixed methods pilot randomly assigned HCPs (*N* = 36) to an intervention (*n* = 17) or control condition (*n* = 19). Implicit and explicit bias measures were administered pretreatment and 3 months postintervention. Differences were analyzed using repeated measures analyses of variance. HCP ratings of virtual patient vignettes depicting Black and White youth with SCD or cancer pain were used to assess differential clinical decision making based on race and diagnosis and analyzed using hierarchical linear mixed model analysis. Focus groups with intervention participants were analyzed using thematic analysis.

**Results:**

No significant differences in scores on bias measures across time, condition, or the Condition × Time interaction were found (all *P* < 0.05). Significant differences in HCP ratings were found between types of HCPs (*P* < 0.001), but no effects were attributable to condition, time, virtual patient race, or diagnosis. Ten themes were extracted regarding the intervention’s format, structure, and content.

**Conclusions:**

This study is the first to evaluate an IPT intervention in pediatric SCD HCPs. HCPs deemed the intervention feasible, acceptable, and impactful and suggested areas for improvement. Future research should refine the intervention to incorporate greater patient involvement and skills practice to improve health outcomes for this underserved population.

## RÉSUMÉ

**Contexte :** Le ​biais implicite des prestataires de soins de santé peut influencer les résultats de santé des jeunes atteints de drépanocytose.

**Buts :** Le but de cette étude était d’évaluer la faisabilité, l’acceptabilité et les effets préliminaires d’une intervention d’individuation et de prise de perspective pour réduire les biais implicites et améliorer la prise de décision clinique en matière de traitement de la douleur chez les professionnels de la santé pédiatrique spécialisés en drépanocytose.

**Méthodes :** Cette étude pilote à méthode mixte a assigné au hasard des professionnels de la santé (N = 36) à un groupe d’intervention (n = 17) ou à un groupe témoin (n = 19). Des mesures de biais implicites et explicites ont été administrées avant le traitement, puis trois mois après l’intervention. Les différences ont été analysées à l’aide d’analyses de variance à mesures répétées. Les évaluations cliniques des professionnels de la santé, fondées sur des vignettes virtuelles de patients représentant des jeunes Noirs et des jeunes Blancs atteints de drépanocytose ou de douleur liée au cancer, ont servi à évaluer la prise de décision clinique différenciée selon la race et le diagnostic. Ces données ont été analysées à l’aide d’un modèle linéaire mixte hiérarchique. Des groupes de discussion ont été menés auprès des participants à l’intervention, et les données ont été analysées à l’aide d’une analyse thématique.

**Résultats :** Aucune différence significative n’a été observée dans les scores sur les mesures de biais en fonction du temps, de l’état ou de l’interaction état × temps (tous les P < 0,05). Des différences significatives ont été trouvées dans les évaluations des professionnels de la santé entre les types de prestataires de soins (P < 0,001), mais aucun effet n’était attribuable à l’état, au temps, à la race virtuelle du patient, ou au diagnostic. Dix thèmes ont été extraits concernant le format, la structure et le contenu de l’intervention.

**Conclusions :** Cette étude est la première à évaluer une intervention d’individuation et de prise de perspective auprès des professionnels de la santé pédiatriques spécialisés en drépanocytose. Les professionnels de la santé ont jugé l’intervention réalisable, acceptable et percutante et ont suggéré des pistes d’amélioration. Les recherches futures devraient affiner l’intervention pour intégrer une plus grande implication des patients et la pratique des compétences, afin d’améliorer les résultats de santé pour cette population mal desservie.

## Introduction

Sickle cell disease (SCD) is a genetic blood disorder that primarily affects individuals of sub-Saharan African descent.^1^ It is the most common genetic disorder in the United States, affecting over 100,000 Americans.^2^ Approximately 2000 babies are born with SCD in the United States each year.^3^ Many patients experience both acute and chronic pain that can begin in childhood, especially during the adolescent and young adult developmental period. Youth with SCD are at high risk for poor health outcomes due to a combination of individual and systemic factors affecting access to quality care, adherence to medical regimens, and biases among health care providers (HCPs: physicians, physician assistants, nurse practitioners, and nurses).^4,5^

There have been increased calls to address implicit bias from HCPs in SCD care, because numerous studies have documented the negative impact of bias on health outcomes.^4,6^ *Implicit bias* describes negative attitudes that occur outside of conscious awareness, whereas *explicit biases* are conscious and deliberate.^4^ In pediatric SCD, HCP bias has been linked to pain undertreatment, low rates of hydroxyurea prescription and referrals for transcranial Doppler screenings, and gaps in treatment during the transition from pediatric to adult care,^4,7,8^ when risk of mortality for patients with SCD increases more than twofold.^5^ Youth with SCD and their caregivers report experiencing racial bias and discrimination from HCPs^9–12^ in the emergency department and outpatient setting, which can harm the patient–provider alliance and exacerbate negative health outcomes.^13^ HCPs who treat SCD often also provide care to individuals with cancer. Interestingly, a qualitative study with Black adults with SCD or cancer found that though both groups reported experiencing delays in treatment and poor communication from HCPs, only those with SCD reported perceived discrimination, being accused of drug-seeking, and feeling unheard by their HCPs.^14^

Previous research has proposed strategies to mitigate implicit bias, such as individuation and perspective-taking. *Individuation* involves focusing on unique personal qualities of the patient rather than group-based characteristics, and *perspective-taking* refers to considering patient experiences from the patient’s point of view.^15,16^ We reviewed the relevant literature and could not find any studies that reported having developed and/or tested an individuation and perspective-taking intervention to address HCP bias.

Previous provider-focused interventions have mostly focused on educational content targeting attitudes and beliefs about caring for patients with SCD.^17,18^ One study tested the impact of an 8-min video intervention compared to no intervention on the attitudes of nurses and house staff toward adults with SCD. The video consisted of a hematologist and three patients with SCD discussing challenges associated with SCD pain care. The results showed decreased negative attitudes and increased positive attitudes toward patients with SCD among those in the intervention condition compared to those in the no intervention control condition.^17^ Another study explored the feasibility and acceptability of a health equity extension for community health care outcomes training. This program consisted of monthly 1-h virtual sessions with didactic presentations and group-based discussion focused on racism and racial justice, as well as strategies to promote change at various levels (e.g., individual, institutional, etc.). Preliminary analyses indicated that the intervention showed promise in increasing HCP self-awareness about implicit bias and racism and in building social justice skills.^18,19^ These studies, though encouraging, have not exclusively targeted pediatric SCD HCPs or produced a validated intervention and are limited in their ability to evaluate the impact of such interventions on HCP behavior, including clinical decision making.

The primary objectives of this mixed methods pilot study were to explore the feasibility, acceptability, and preliminary impact of an individuation and perspective-taking (IPT) intervention intended to decrease implicit bias and improve the effectiveness of pain treatment clinical decision making in pediatric SCD HCPs. Previous research has that proposed training based on IPT may mitigate bias.^15,16^ We hypothesized that, compared to HCPs in a didactic information control condition, HCPs assigned to the IPT intervention would demonstrate (1) lower levels of implicit racial bias as assessed by the Race Implicit Association Test (IAT^20^) and explicit bias as assessed by an explicit SCD bias measure^21^ and (2) more effective pain treatment clinical decision making (i.e., consistent with published guidelines for care) as assessed by ratings on virtual patient vignettes. A secondary objective was to explore differences in pain treatment decisions for virtual patients with SCD versus cancer pain, given that both diagnoses are treated by pediatric HCPs within the hematology/oncology subspecialty. Based on previous research,^14^ we expected that HCPs would make more effective pain treatment clinical decisions for virtual patients with cancer. Finally, we conducted focus groups with HCPs in the intervention arm to elicit their perspectives on the feasibility, acceptability, and preliminary impact of the IPT intervention. We hypothesized that HCPs’ responses would largely support the feasibility and acceptability of the intervention.

## Methods

This explanatory sequential mixed methods pilot study was conducted in a web-based format at a mid-sized, freestanding children’s hospital in the Northeastern United States. Approval from the Connecticut Children’s Institutional Review Board (IRB) was obtained prior to all study procedures (IRB No. 21-036). The research design of the quantitative phase was a randomized pilot and the qualitative phase used a descriptive–interpretative design. Study goals were to understand complex phenomena (i.e., implicit and explicit bias) and measure change over time. The objective of the quantitative phase was influence of the intervention on HCP bias and pain treatment clinical decision making, and exploration of the feasibility, acceptability, and overall impact of the intervention was the objective of the qualitative phase. Mixed methods were used for the purpose of participant enrichment^22^ to determine whether the intervention contributed to change (e.g., less bias) and how it impacted participants. IPT is intended to impact HCPs’ communication with patients to reduce biases. Given that this study was a pilot study, it was not registered in a clinical trails database.

### Participants

A sequential nested sampling design was used in this study. Participants were 36 physicians, advanced practice providers, and registered nurses who provide care to youth with SCD in the inpatient and/or outpatient (i.e., clinic-based) setting. Advanced practice providers were physician assistants and nurse practitioners. Participants consisted of primarily White (*n* = 33; 91.6%), cisgender females (*n* = 32; 88.9%) with a mean age of 38.6 years. Participants included physicians (*n* = 10; 27.8%), nurses (*n* = 11; 30.6%), advanced practice providers (*n* = 8; 22.2%), and medical trainees (*n* = 7; 19.4%). On average, most participants (*n* = 21; 58.3%) reported having at least 6 years of experience caring for patients with SCD. See Table 1 for participant characteristics.

**Table 1.** Provider characteristics (*N* = 36).

Characteristic	*N* (%)		
	Total	Intervention	Control
Gender identity			
Cisgender female	32 (88.9)	14 (38.9)	18 (50.0)
Cisgender male	4 (11.1)	3 (8.3)	1 (2.8)
Race			
White	31 (86.1)	14 (38.9)	17 (47.2)
Asian	3 (8.3)	2 (5.6)	1 (2.8)
More than one race	1 (2.8)	1 (2.8)	0 (0.0)
Other	1 (2.8)	0 (0.0)	1 (2.8)
Ethnicity			
Not Hispanic/Latino	35 (97.2)	16 (44.4)	19 (52.8)
Hispanic/Latino	1 (2.7)	1 (2.8)	0 (0.0)
Profession			
Physician	10 (27.7)	2 (5.6)	8 (22.2)
Nurse	11 (03.6)	7 (19.4)	4 (11.1)
App*	8 (22.2)	4 (11.1)	5 (13.9)
Medical trainee	7 (19.4)	4 (11.1)	3 (8.3)
Primary work location			
Hematology/oncology clinic	17 (47.2)	8 (22.2)	9 (25.0)
Inpatient unit	7 (19.4)	3 (8.3)	4 (11.1)
Emergency department	8 (22.2)	4 (11.1)	4 (11.1)
Other	4 (11.1)	2 (5.6)	2 (5.6)

*advanced practice provider*M*_age_ = 38.6 years (SD 9.5).

Participants were recruited from the Hematology/Oncology and Emergency Medicine departments and from inpatient medical/surgical units. Recruitment occurred by contacting department heads and administrative staff to share study information with eligible participants via team huddles, e-mail, and IRB-approved flyers posted in staff breakrooms. Inclusion criteria were (1) older than 18 years of age, (2) provided direct patient care for at least 10 h per week during the study period, (3) licensed or certified health care professional or medical trainee, and (4) English fluency and ability to provide informed consent.

**Figure 1.**
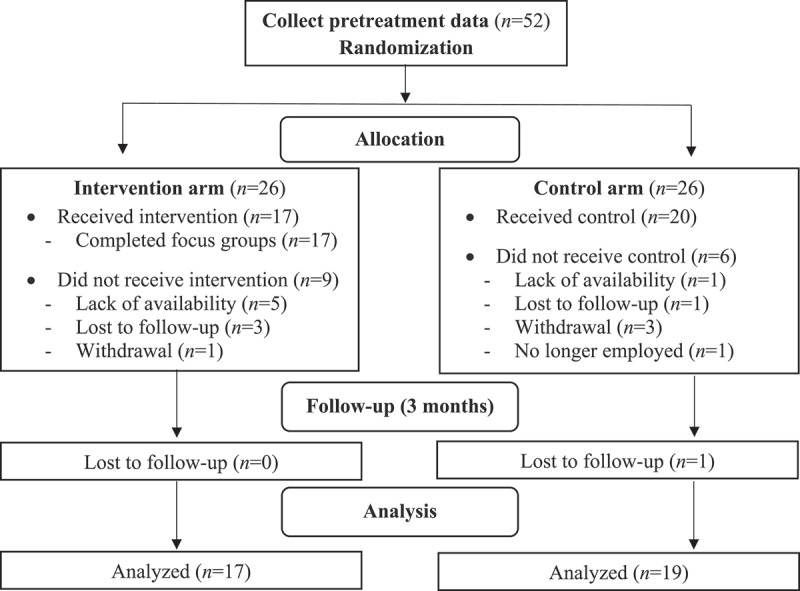
Participant recruitment, retention, and attrition.

A total of 52 participants initially expressed interest in study participation, completed pretreatment measures, and were randomly assigned to the intervention or control condition. However, 15 of these participants (29%) either were unable to be scheduled for the IPT intervention or control session due to lack of availability (*n* = 6), were lost to follow-up (*n* = 4), withdrew from the study (*n* = 4), or were no longer employed at the institution (*n* = 1). Participants were emailed up to three times to schedule their session. Those who did not respond were considered lost to follow-up. Additionally, one participant was lost to follow-up at 3 months postintervention (never responded to emailed postintervention survey links), resulting in a total *N* = 36 (*n* = 17 intervention participants, *n* = 19 control participants) for all analyses. See Figure 1 for a diagram of participant recruitment, retention, and attrition. Five focus groups (3–4 participants in each) were completed with all 17 intervention participants.

### Measures

#### Demographic Questionnaire

Participants completed a demographic questionnaire pretreatment that assessed their age, race/ethnicity, gender identity, role, primary work location, years of experience in their current profession, and years of experience treating patients with SCD.

#### Race IAT

Participants’ implicit attitudes about race were assessed with the Race IAT.^20^ The Race IAT has been widely used as a measure of implicit racial bias,^20,23,24^ including among HCPs.^25^ On the Race IAT, participants categorize—as quickly as possible without making errors—facial images as depicting a Black or White person and evaluative words as good or bad (e.g., “pleasure” is a good word and “awful” is a bad word). Separate computer keys are used to make these categorical judgments. In one trial, participants were instructed to use the same key to indicate a White face or good word and a different key to indicate a Black face or bad word. In a second (reverse) trial, participants were instructed to use the same key to indicate a White face or bad word and a different key to indicate a Black face or good word. Faster responses to the White/good + Black/bad pairings than to the White/bad + Black/good pairings indicated stronger positive bias associated with White over Black people^26^ and vice versa. Participants received a *D* score ranging from −2 to +2, with positive values indicating more positive bias associated with White people and negative values indicating more positive bias associated with Black people.^26^ A common rule of thumb is that values of 0.15 to 0.34 indicate slight positive bias, 0.35 to 0.64 moderate positive bias, and ≥0.65 strong positive bias.^26^

Two Race IATs were coded into the Qualtrics survey for the purpose of this study, one with adult faces and the other with child faces. At each time point (pretreatment and 3-month follow-up), participants completed the Race IAT twice, once with the adult faces and once with the child faces, which yielded two separate scores. The order in which the two versions were presented was randomized to reduce testing bias.

#### Virtual Patient Vignettes

**Figure 2.**
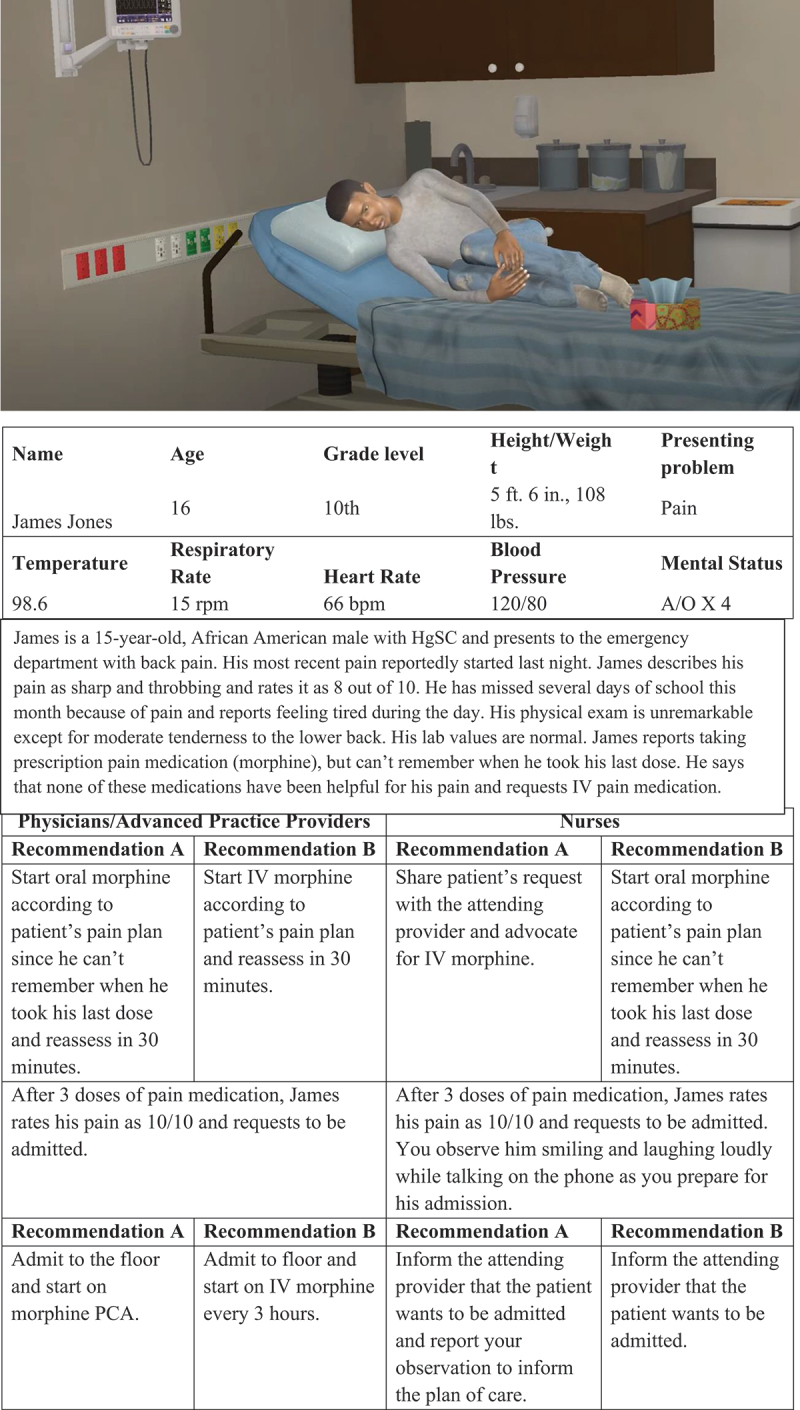
Virtual patient vignette, Black adolescent with sickle cell pain.

**Figure 3.**
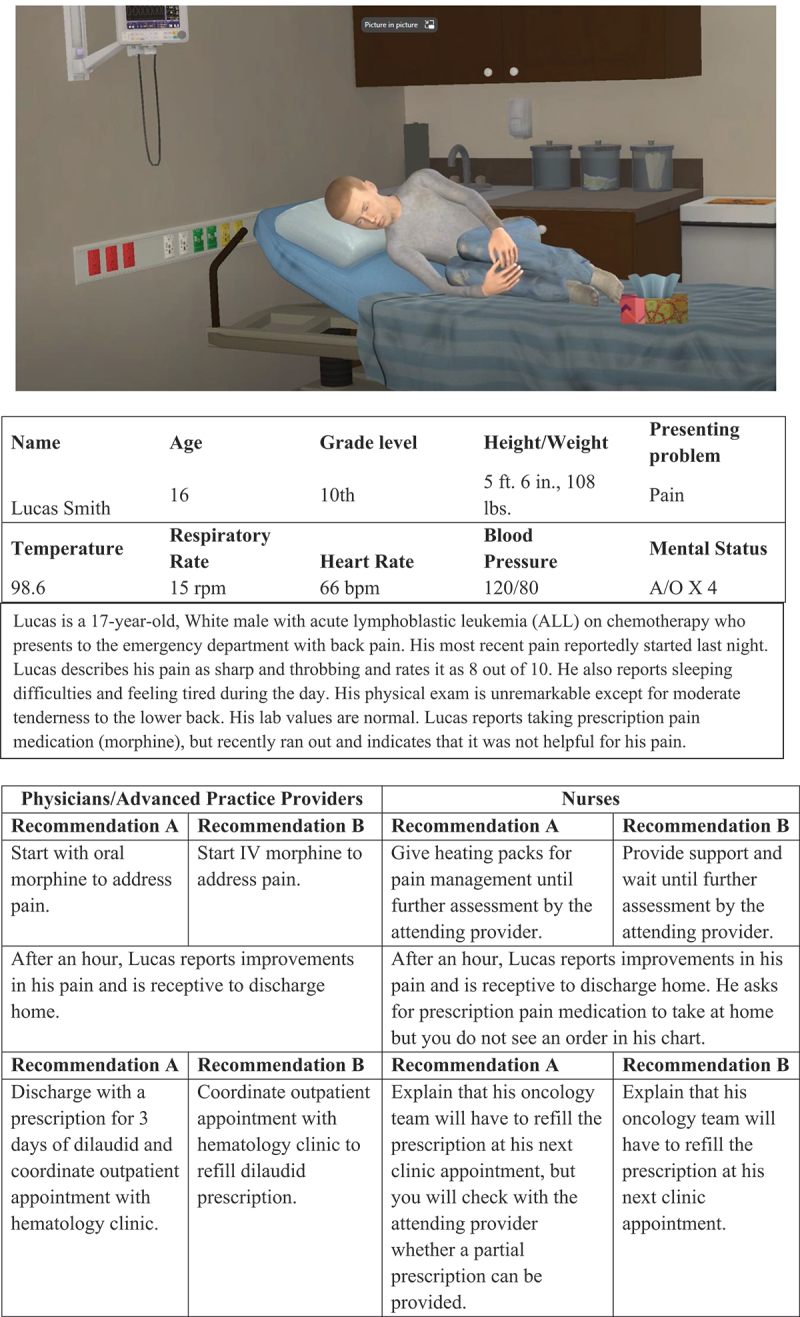
Virtual patient vignette, White adolescent with cancer pain.

Virtual patient vignettes (see Figures 2 and 3) were developed to assess HCP pain treatment clinical decision making.^27^ Virtual patient technology has been used in previous studies to assess the impact of patient demographic variables, including race/ethnicity, on pain assessment, clinical decision making, and treatment disparities.^28–30^ Virtual patient vignettes for this study, including treatment questions and response options, were systematically modeled after those used in previous research^21^ and pilot-tested in pediatric hospitalists and medical trainees.^27^

Participants were shown four unique, animated virtual patient vignettes (two Black male adolescents with SCD and one Black and one White male adolescent with acute lymphocytic leukemia). Each video was 60 s long and showed virtual patients exhibiting pain behavior in an emergency department hospital room. The animations were accompanied by text descriptions of the patient and presenting problem, along with physiological data (e.g., temperature, heart rate).

For each vignette, providers were asked to rate their agreement with each of two pairs of treatment plans, one pair pertaining to the initial stage of the encounter and another pair of treatment plans pertaining to treatment once the patient had been evaluated. For each pair of plans at each stage, one of the pair represented a preferred treatment plan (i.e., one that was consistent with published guidelines for care or “best practices”) and the other plan deviated from guidelines. Providers rated each plan for each stage of the encounter on a scale from 1 = *strongly disagree with this plan* to 5 = *strongly agree with this plan*. Thus, each provider had four scores for each of the four vignettes. A “best practice score” for each vignette was calculated by taking the mean of the scores on the two preferred treatment plans, with higher scores indicating stronger agreement with treatment guidelines for care.

For example, when presented with a virtual patient with SCD (“James”; see Figure 2), at the initial stage of the encounter, physicians rated their level of agreement with (1) start intravenous morphine according to patient’s pain plan and reassess in 30 min or (2) start oral morphine according to patient’s pain plan because he cannot remember when he took his last dose and reassess in 30 min. After rating these decisions, physicians were presented with additional written information regarding the virtual patient’s status after having initial treatment (“After three doses of pain medication, James rates his pain as 10/10 and requests to be admitted”) and a second pair of treatment plans was presented, to which they indicated their agreement with (3) admit to the floor and start on morphine patient-controlled analgesia pump or (4) admit to the floor and start on intravenous morphine every 3 h.

Nurses were presented with a different set of treatment plans for each vignette. When presented with the same virtual patient with SCD (“James”), nurses first rated their agreement with the following treatment plans: (1) Share patient’s request [for intravenous pain medication] with the attending provider and advocate for intravenous morphine or (2) start oral morphine according to patient’s pain plan because he cannot remember when he took his last dose and reassess in 30 min. Additional written information was presented to nurses after the virtual patient had received initial treatment (“After three doses of pain medication, James rates his pain as 10/10 and requests to be admitted. You observe him smiling and laughing loudly while talking on the phone as you prepare for his admission”). Nurses then rated their agreement with (3) inform the attending provider that the patient wants to be admitted and report your observation to inform the plan of care or (4) inform the attending provider that the patient wants to be admitted.

#### Explicit SCD Bias

Explicit bias against youth with SCD was assessed by using a measure of explicit bias from a previous study,^21^ which was based on other validated measures of explicit bias.^31^ Participants rated their level of agreement on a 5-point Likert scale ranging from 1 = *strongly disagree* to 5 = strongly agree with three statements that reflect negative stereotypes of patients with SCD: (1) Treating youth with SCD is more challenging than treating youth with other illnesses, (2) youth with SCD seem to present with less urgent issues than youth with other illnesses, and (3) youth with SCD are often less compliant than youth with other illnesses. The item scores were summed to create an explicit bias total score, ranging from 3 to 15, with higher scores indicating stronger agreement with SCD stereotypes.

### Procedures

Prospective participants were emailed a unique link to study measures. Measures were administered at two time points (pretreatment and 3 months postintervention) via Qualtrics, a web-based survey tool. Measures were administered at 3-month follow-up for the following reasons: (1) to reduce testing effects from completing the Race IATs, (2) to reduce participant burden, and (3) to determine retention of the IPT skills, given that few implicit bias interventions facilitate sustained change over time.^32^ Participants provided informed consent electronically by selecting “yes” prior to beginning the survey. Our study procedures are at increased risk for socially desirable and biased responding from participants. To minimize this, we received IRB approval to use mild deception/incomplete disclosure during the consent process, such that the study was described as focusing on clinical decision making rather than implicit bias. Measures were completed at participants’ convenience by a given deadline. After completing pretreatment measures, participants were given a study ID and randomly assigned to the intervention or control condition by C.B.T. Randomization was conducted with a random integer sequence generator (RANDOM.ORG) that generates randomness based on atmospheric noise.^33^ Integers corresponded to participant study ID numbers and the sequence was produced as two separate even columns for either the intervention or control group. Only C.B.T. had access to participants’ assignments; all other members of the research team were blinded to participant assignment.

Sessions were conducted in small groups and scheduled based on participants’ availability, which resulted in five intervention sessions (three to four participants in each) and six control sessions (three to four participants in each). There was one more control than intervention session due to differences in the overall group size (*n* = 20 control participants versus *n* = 17 intervention participants). Intervention participants were also invited to participate in focus groups immediately postintervention to explore the feasibility, acceptability, and overall impact of the intervention. Focus groups were conducted as soon as possible following participants’ completion of the intervention to facilitate recall and retention. To minimize social desirability bias, focus group participants were told that they would remain anonymous, their responses would be kept private and confidential, and they were not required to turn on their video cameras during focus groups sessions. Three months postintervention, all participants were emailed a link up to three times to complete posttest measures via Qualtrics. Following the completion of posttest measures, participants were emailed a form describing the use of deception/incomplete disclosure, alongside a short video of the principal investigator (S.S.M.) explaining why this was used. Participants were compensated with a $25 VISA gift card for completing the pretreatment survey, a $35 VISA gift card for participating in the intervention, a $40 VISA gift card for participating in focus group sessions (if applicable), and a $50 VISA gift card for completing the 3-month follow-up survey.

#### Intervention and Control Conditions

The intervention group participated in a 90-min live virtual session conducted via Zoom using didactic and experiential learning that heavily focused on the use of two cognitive strategies that have been previously proposed to reduce bias among HCPs (individuation and perspective-taking).^15^ The IPT intervention was developed in consultation with experts in implicit bias and SCD pain care and conducted by an expert in the field (not an author or member of the research team). The content consisted of a brief didactic presentation on the cycle of socialization, neurobiology of implicit bias, and history of medical racism relevant to the SCD population, followed by an in-depth explanation of the IPT cognitive strategies and practice using these skills with publicly available SCD patient videos (https://www.youtube.com/watch?v=yCOvkOCaaSk). The control condition was exposed to a 60-min prerecorded virtual session about best practices in pediatric SCD pain management delivered by a medical expert in pediatric SCD pain (W.T.Z.).

#### Focus Groups

Intervention group participants were invited to participate in a 30-min virtual focus group postintervention via Zoom. When possible, focus groups were conducted immediately postintervention to facilitate participant recall and retention. Five focus groups of three to four participants each were conducted by trained research personnel (C.B.T. and S.D.) using a semistructured format and questioning route, which is the planned sequence of questions, beginning with broad questions and becoming increasingly focused on the construct(s) of interest. Focus group questions centered on the intervention’s feasibility (e.g., format, length, etc.), acceptability (e.g., relevance to participants’ work), and overall impact (e.g., interest in maintaining IPT skills and applying them to patient care) and elicited feedback on areas for improvement. Questions included “Do you think the intervention was relevant to your clinical work with patients with sickle cell disease? Why or why not?” (acceptability) and “What did you like/dislike about the structure and format of the intervention?” (feasibility). The average duration of focus groups was approximately 25 min. Each focus group was audio recorded, deidentified, and transcribed using a secure transcription service. Transcriptions were then reviewed for accuracy by other members of the research team prior to conducting a thematic analysis.

### Data Analysis

#### Quantitative

The Statistical Package for Social Sciences v20 was used for all statistical analyses. Descriptive statistics were calculated for participant demographics. Frequency tables, along with the means and standard deviations, were calculated for the Race IAT scores with adult and child faces and responses to the explicit SCD bias measure at pretreatment and 3 months postintervention. Means and standard deviations were also calculated separately for physician/advanced practice provider and nurse virtual patient vignette best practice ratings.

Race IAT scores for both adult and child faces were analyzed separately using mixed effect repeated measures analyses of variance (ANOVAs). Analyses were performed to examine the main effects of condition (control versus intervention), time (pretreatment and 3 months postintervention), and the Condition × Time interaction on Race IAT D scores.

As described above, for each virtual patient, providers made two agreement ratings (initial stage and following evaluation). The “best practice score” was calculated as the mean of the agreement ratings on the two preferred treatment plans. These best practice scores were analyzed using a hierarchical linear mixed model analysis. The scores for multiple patients (the two patients with SCD and the two with cancer) were nested within subject within time. The analysis modeled the main effects of condition (control versus intervention), time (pretreatment versus 3 months follow-up), provider type (nurse versus physician or advanced practice provider), the race of the patient (Black versus White), and the diagnosis of the patient (SCD versus cancer), plus the interactions of Condition × Time, Condition × Patient race, Condition × Patient diagnosis, and Condition × Time × Patient race. We were thus able to determine whether HCPs’ clinical decision making differed by treatment, patient race, or patient diagnosis and by whether the HCP was a prescriber (physician or advanced practice provider) or a nurse, as well as whether any of these interacted with other status variables.

Finally, explicit bias scores were analyzed using repeated measures ANOVA. Bias scores were analyzed for differences attributable to condition, time, provider type, Condition × Time, Provider type × Time, and Condition × Time × Provider type.

#### Qualitative

An inductive thematic analysis^34^ of focus group transcriptions was conducted using HyperRESEARCH v 4.5.4. HyperRESEARCH was selected for qualitative analyses due to its affordability, user-friendly interface, enhanced collaboration tools, extensive customer support, and authors’ previous experience using this software. Two study personnel (C.B.T. and S.D.) reviewed the focus group transcripts independently and created codebooks to identify key themes extracted from the focus group sessions. The personnel met to discuss and compare findings. Coding differences were resolved through consensus. A master codebook was then created to summarize key themes that were extracted from the data. Results were triangulated using a brief, five-item intervention feedback form that evaluated participants’ expectations and perceived utility of the IPT intervention and whether they would recommend it to a friend. Participants were also asked to write down three things they learned from the intervention and what they would change to improve the intervention.

### Data Validation/Legitimation

#### Quantitative

Threats to internal validity were addressed by including a control group and randomly assigning participants to a condition. Threats to external validity were addressed by including clear inclusion/exclusion criteria, using a pre/post design to reduce testing effects, and using mild deception/incomplete disclosure to minimize social desirability bias.

#### Qualitative

Threats to data trustworthiness and credibility were addressed by triangulating themes from focus groups with the intervention feedback form. Participants’ responses were summarized at the end of each focus group to elicit feedback/corrections further supported data credibility.

## Results

### Quantitative Data

There were no missing data from survey and Race IAT responses for any participant between pretreatment and follow-up. Means and standard deviations for all outcome measures are presented by treatment condition (intervention versus control) across the two time points (pretreatment and 3-month follow-up) in Table 2.

**Table 2.** Means and standard deviations for primary outcome measures by treatment condition.

Measure	Pretreatment, mean (SD)		3 Months postintervention, mean (SD)	
	Intervention	Control	Intervention	Control
Race Implicit Association Test				
Adult faces	0.45 (0.34)	0.45 (0.31)	0.49 (0.29)	0.37 (0.37)
Child faces	0.46 (0.25)	0.52 (0.24)	0.38 (0.30)	0.46 (0.34)
VP vignettes				
Physicians/App*	3.34 (1.39)	4.13 (0.49)	4.18 (0.57)	3.98 (0.71)
Nurses	3.80 (0.49)	3.38 (0.65)	3.27 (0.36)	3.17 (0.14)
SCD explicit bias	6.76 (2.02)	6.95 (1.93)	7.35 (1.90)	7.32 (2.34)

*advanced practice provider

#### Race IAT

Categorical preference tendencies for White versus Black faces are shown in Table 3. Participants demonstrated a clear pro-White bias for both adult and child faces pretreatment. For adult faces, 80.6% of our sample demonstrated a pro-White bias, with the largest percentage demonstrating a *strong* pro-White bias (36.1%), whereas only 16.7% demonstrated no bias. For child faces, 94.4% of participants demonstrated a pro-White bias, with the largest percentage (47.2%) demonstrating a *moderate* pro-White bias and only 5.6% demonstrating no bias. At 3 months postintervention, participants continued to show pro-White biases for both adult and child faces. For adult faces, 77.8% of our sample demonstrated a pro-White bias, with the largest percentage being a *moderate* pro-White bias (38.9%) and 16.7% showing no bias. For child faces, 80.1% of participants demonstrated a strong pro-White bias, with the largest percentage being a *strong* pro-White bias (30.6%) and 13.9% showing no bias.

**Table 3.** Categorical tendencies indicated by the race implicit association test.

Interpretation	Adult faces				Child faces			
	Pretreatment		3 Months postintervention		Pretreatment		3 Months postintervention	
	*N*	%	*N*	%	*N*	%	*N*	%
Slight pro-White bias	6	16.7	6	16.7	8	22.2	8	22.2
Moderate pro-White bias	10	27.8	14	38.9	17	47.2	10	27.8
Strong pro-White bias	13	36.1	8	22.2	9	25.0	11	30.6
No bias	6	16.7	6	16.7	2	5.6	5	13.9
Slight pro-Black bias	1	2.8	2	5.6	0	0.0	2	5.6
Total pro-White bias	29	80.6	28	77.8	34	94.4	29	80.1

Results of the repeated measures ANOVAs on IAT *D* scores indicated that there were no main effects for intervention condition or time on IAT scores of adult faces or child faces, nor was there a Condition × Time interaction (see Table 4).

**Table 4.** Summary of results of repeated measures analysis of variances of race implicit association test *D* scores for adult and child faces.

	Treatment condition										
	Control (*n* = 19)				Intervention (*n* = 17)				Effect tested^a^		
	Pretreatment		3 Months postintervention		Pretreatment		3 Months postintervention				
Target face	Mean	SD	Mean	SD	Mean	SD	Mean	SD	Treatment	Time	Treatment × Time
Adult	−0.45	0.31	−0.37	0.37	−0.45	0.34	−0.49	0.29	0.45	0.06	0.85
Child	−0.52	0.24	−0.46	0.35	−0.46	0.25	−0.38	0.29	1.63	0.87	0.26

^a^Shown are *F* values. *df* = 1/34 for each effect.

#### Virtual Patient Vignettes

Pretreatment and 3 months postintervention, all participants rated the preferred treatment plans higher than the alternative for each of the four virtual patients. Table 5 shows the results of the linear mixed model analysis of treatment plan agreement ratings, aggregated over all four virtual patients. As seen in the table, there were no effects on treatment plan ratings attributable to condition (intervention or control), time, virtual patient race, or diagnosis. There were differences seen between types of providers, such that physicians and advanced practice providers tended to indicate more agreement with the preferred treatment plans than did the nurses (*M* for physicians and advanced practice providers = 3.94, SD 0.95; *M* for nurses = 3.19, SD 0.89).

**Table 5.** Results of linear mixed model analysis of providers’ scores on vignette treatment plans.

Effect tested	*df*	F
Condition (control versus intervention)	1/33	0.41
Time (pretreatment versus 3-month posttreatment)	1/34	0.14
Provider type (nurse versus physician/App**)	1/33	26.57*
Patient race (Black versus White)	1/34	1.62
Patient diagnosis (SCD versus cancer)	1/34	0.29
Condition × Time	1/34	2.00
Condition × Patient race	1/34	2.00
Condition × Patient diagnosis	1/34	0.10
Condition × Time × Patient race	2/34	0.07

*P < 0.001.**advanced practice provider

#### SCD Explicit Bias Measure

All participants demonstrated low levels of agreement with negative stereotypes about youth with SCD pretreatment (*M* = 6.86, SD 1.94). Just under half agreed that treating sickle cell disease is more challenging than treating other illnesses (*n* = 16, 43.2%). Only one agreed that patients with sickle cell disease are less compliant than youth with other illnesses (2.7%), and only five (13.5%) agreed with the statement that patients with sickle cell disease present with less urgent issues than patients with other illnesses. At follow-up, scores remained similar (*M* = 7.33, SD 2.11). Approximately half agreed that treating sickle cell disease is more challenging than treating other illnesses (*n* = 18; 48.6%). Only one still agreed that patients with sickle cell disease are less compliant than youth with other illnesses (2.8%), and eight agreed with the statement that patients with sickle cell disease present with less urgent issues than patients with other illnesses (22.2%).

Results of the repeated measures ANOVA indicated that the intervention and control groups did not significantly differ in their scores on the SCD explicit bias measure, nor were there any effects on explicit bias attributable to time or provider type (see Table 6).

**Table 6.** Results of repeated measures analysis of variances of providers’ scores on the sickle cell disease explicit bias measure.

Effect tested	*df*	F
Condition (control versus intervention)	1/33	0.18
Time (pretreatment versus 3-month posttreatment)	1/34	2.05
Provider type (nurse versus physician/App*)	1/33	0.41
Condition × Time	1/34	0.25
Condition × Provider type	1/34	0.77
Provider type × Time	1/34	0.63
Condition × Time × Provider type	1/34	0.19

*advanced practice provider

### Qualitative Data

Analysis of transcripts of focus groups with intervention participants identified ten themes related to the feasibility, acceptability, and impact of the intervention, as well as specific recommendations on strategies for improvement. Themes included logistical barriers, virtual format, well-organized (feasibility), relevant/applicable to work, training requirement, similar to previous training (acceptability), patients’ lived experiences, skills practice and mindset, more practice with skills, and patient involvement (strategies for improvement; see Table 7).

**Table 7.** Themes from provider focus groups.

Content area	Generated themes
Feasibility	Logistical barriers: Participants reported concerns about time commitment due to the length of the intervention. Virtual format: Participants reported the virtual format of the intervention was beneficial for their participation.
Acceptability	Well-organized: Participants reported that the intervention was well organized and delivered by a knowledgeable presenter. Relevant/applicable to work: Participants reported that the intervention was highly applicable to their jobs. Training requirement: Participants generally reported that the intervention should be an employment requirement. Similar to previous training: Participants indicated the didactic portion of the intervention was a good reminder of previous trainings (positive) but also redundant (negative).
Impact	Patients’ lived experiences: Participants described value in the examples of patients’ lived experiences incorporated into the intervention (i.e., patient videos). Skills practice and mindset: Participants discussed value in learning and practicing the IPT skills and described how these skills can impact of their mindset during patient encounters.
Suggestions for improvement	More practice with skills: Participants suggested including additional time to practice individuation and perspective-taking skills. Patient involvement: Participants suggested that more stories from patients with SCD should be incorporated into the intervention.

#### Feasibility

Focus group participants agreed that the IPT intervention was well organized and that the presenter was highly knowledgeable. One nurse shared, “I thought the virtual format worked well, and I think [she] was a really great speaker. I liked the way that she presented and incorporated us into the discussion” (Participant 28, hematology/oncology nurse, 6–10 years of experience). Though there was general agreement that the intervention should be required for all clinical staff, many participants highlighted logistical barriers to offering this education to HCPs at all levels of care (e.g., inpatient, outpatient, etc.). They expressed concerns about its length, noting that 90 min could be difficult to fit into busy clinical schedules. One nurse indicated, “I think this is important work and, you know, any of the training that we do is certainly important. It’s just a matter of carving out time or finding staffing to make sure that from a nursing standpoint, we can staff that for a while” (Participant 38, inpatient nurse, <1 year of experience). The time requirement was the most consistent obstacle identified.

To address logistical issues, participants recommended scheduling the intervention to coincide with existing trainings/meetings, such as during employee orientation/onboarding, grand rounds, lunch sessions, and/or required annual education. They also emphasized the need for institutional support for the training. One medical trainee said, “It would need to be like an evening or put into one of our didactic slots. … It would need to fit into a space that is already reserved for some type of educational session” (Participant 48, pediatric resident).

Participants recommended that the intervention stay virtual, acknowledging that virtual trainings are easier for staff to attend and thus may help overcome some of the logistical challenges. Some participants noted that this was a very personal training with potentially sensitive discussion, which could be even more effective in person. One nurse noted, “I really did like the small group. I was a little nervous because it kind of forced you to talk more but also I felt more comfortable because I could see and I knew … who I was talking to” (Participant 28, hematology/oncology nurse, 6–10 years of experience). A few participants suggested a hybrid model but indicated that a hybrid approach may introduce still more logistical hurdles. One nurse observed, “The virtual [format] is nice because you can always get everyone in the same room at the same time” (Participant 20, hematology/oncology nurse, >15 years of experience).

#### Acceptability

Most focus group participants found the IPT intervention to be relevant and applicable to their job and a good reminder of prior trainings. A nurse reflected, “I see the importance and I think that this is very relevant” (Participant 38, inpatient nurse, <1 year of experience). Though a few participants found the content redundant or too similar to previous instruction, many articulated that the intervention was important and valuable to their work. Some noted that they had previously learned a lot about racism and discrimination and highlighted the importance of demonstrating how implicit biases directly impact patient care, specifically for patients with SCD. They also noted that the patient videos helped keep them engaged with the content. One medical trainee shared, “I thought that the smaller group was really helpful just because … it’s really intimidating to talk in front of a lot of people about things that you may be feeling that are more personal” (Participant 51, pediatric intern).

#### Impact

For many, the most impactful and helpful parts of the intervention were the videos of children and families talking about their experiences with SCD, which HCPs used to practice the IPT skills. The videos showed the real experiences of patients and caregivers, in their own words, and helped HCPs understand the clear value of seeing patients as individuals (individuation) and understanding the interactions from the patient’s perspective (perspective-taking). For example, one nurse noted, “My favorite was the [video]. Hearing … from the patients themselves, what they go through. We see the kids when they come into the hospital for crisis; we don’t see them when they’re doing well at home. So that was really helpful for me” (hematology/oncology nurse, 6–10 years of experience). Others noted that, until recently, HCPs referred to patients with sickle cell disease as “sicklers,” thus failing to see them as individuals and reducing them to their disease. The videos helped reinforce that different patients deal with pain differently and that HCPs often see patients on their worst days. Focus group participants highlighted the value of both learning and practicing the IPT skills. Several indicated that there was not enough time at the end of the training to practice these skills. They noted that too much time was spent laying out the issue and providing background about implicit bias; they would have preferred to have had more time to practice the IPT skills.

### IPT Skills

Many participants reflected on the particular skills associated with the IPT intervention and saw clear pathways for how these skills could inform their clinical work. Some identified how this training could improve their effectiveness as HCPs. One medical trainee observed, “It’s always good to be reminded, to take a step back and think about what everyone else is going through and kind of what might be contributing to why [the patient’s] acting a certain way” (Participant 45, pediatric resident). Others articulated how to apply the lessons and skills to their current work, laying out easy steps that can help reinforce the goals of individuation and perspective-taking. One nurse practitioner reflected:

Before you go into the room, trying to remember something that you know about them that’s totally separate from why they’re in the hospital. I think trying to connect—I think that’s such an important tool and we know that patients do better when they feel connected and trust their providers. And we do a better job of taking care of patients when we feel connected to our patients and we care about them. So really trying to remember that stuff. It’s not just about knowing that they are an individual but connecting to them. (Participant 1, hematology/oncology nurse practitioner, 6–10 years of experience)

Many participants discussed the importance of slowing down and taking a breath before interacting with their patients, taking a moment to acknowledge the pain and frustration that their patients and families are experiencing, and also using that time to remind themselves that they are real people, not a diagnosis.

#### Suggested Improvements

Focus group participants had specific suggestions for improving the IPT intervention. One suggestion was to include patients with SCD in the intervention to enhance its impact by hearing directly from patients. One HCP suggested, “Like I said, I thought the cases were helpful, and seeing the kids’ videos were helpful. It’d be really impactful if you had an actual sickle cell patient to talk to us live via Zoom, for like five minutes” (Participant 18, emergency department physician, 10–15 years of experience). A nurse practitioner indicated, “It would have also been helpful to hear the patient perspective of how providers can be helpful in their communication” (Participant 9, hematology/oncology nurse practitioner, >15 years of experience).

Participants also suggested incorporating tips on how to ask different kinds of questions into their clinical encounters, beyond the standard medical questions or questions about their pain, thus building on the goals of individuation, or seeing each patient as an individual. The vast majority of participants recommended shortening the didactic portion of the presentation in favor of more time to practice skills. Some even suggested practice changes as a result of the intervention, such as beginning each day and/or each team meeting with a short video or story of a patient’s perspective to get in the right mindset for the day ahead.

### Data Integration and Triangulation

Quantitative and qualitative data were integrated using a triangulation strategy, which combines multiple perspectives for a comprehensive understanding of the research question. The qualitative and quantitative results were compiled and integrated to draw overall conclusions (see Table 8). First, we compared study surveys with focus group transcripts from intervention participants to identify patterns of convergence and divergence, which allowed us to obtain a more detailed and holistic evaluation of the IPT intervention. Results of the intervention feedback form corresponded to themes related to the intervention’s feasibility, acceptability, and overall impact. Specifically, both the feedback form and the focus group results emphasized the importance of addressing bias in providing care and the relevance of the intervention. Additionally, suggested areas for improvement from both the intervention feedback form and focus group themes included incorporating real patient stories. Though we did not find any significant differences pre- and posttreatment on the Race IAT, virtual patient vignettes, or SCD explicit bias measure as a function of the intervention, the combined quantitative findings led to inferences that were complementary and reinforcing to the qualitative findings. HCPs exhibited mild to moderate levels of pro-White bias, supporting the importance of bias in care provision, but clinical judgments were not affected by race or diagnosis of the patient. The intervention was well received but did not impact implicit bias or clinical decision making. Thus, though perceived as relevant, the intervention was subject to improvement, in both form (e.g., duration) and content (e.g., inclusion of more real-life patient stories).

**Table 8.** Joint display integration of quantitative and qualitative findings.

Area of focus	Quantitative data				Summary derived from quantitative findings	Qualitative data
	Int. feedback form	Race IAT	Virtual patient vignettes	SCD explicit bias		Focus group themes
Feasibility	71% agreed that intervention met expectations	N/A	N/A	N/A	Intervention is feasible	Logistical barriers Virtual format
Acceptability	65% agreed that intervention useful for professional development and clinical practice	N/A	N/A	N/A	Intervention is acceptable	Well-organized Relevant/applicable to work Training Requirement Similar to previous training
Impact	71% agreed that they would recommend intervention to others; improvements needed to increase impact	Mild to moderate levels of implicit bias	Race/diagnosis showed no impact on pain treatment clinical decisions	No impact on negative SCD stereotypes	Intervention lacks impact on bias	Patients’ lived experience Skills practice and mindset
Suggested improvements	Incorporate real patient stories	N/A	N/A	N/A	Inclusion of patient stories well received	More practice with skills Patient involvement

## Discussion

This mixed methods pilot study is the first to evaluate a theoretically and empirically informed intervention to foster individuation and perspective-taking among pediatric HCPs caring for youth with SCD. In this initial evaluation of the intervention we compared HCPs randomized to one of two conditions (intervention versus control) on measures of implicit (Race IAT) and explicit (SCD explicit bias measures) racial bias and HCP pain treatment decisions for virtual patient vignettes at two time points (pretreatment and 3-month follow-up). Though the intervention and control conditions did not significantly differ in implicit or explicit bias or pain treatment decision making, these findings are preliminary. Enhancements to the IPT intervention may make it more efficacious in future studies. HCPs who participated in the intervention nevertheless found it valuable, as evidenced by focus group findings indicating that the IPT intervention was deemed to be feasible, acceptable, and impactful. Focus group participants also had several suggestions for improving the intervention content and format, which could ultimately have implications for addressing provider bias and pain treatment decision-making.

Our sample of HCPs who received the intervention provided positive feedback overall on its format, content, and impact. They reported some overlap with previous implicit bias training but indicated that the focus on learning the IPT skills was novel and highly valued. As such, they recommended shortening the didactic portion of the intervention and allowing more time for IPT skills practice with stories from patients with SCD. Moreover, the inclusion of patient stories to practice these skills was a unique aspect of the IPT intervention, and participants suggested greater patient involvement as one strategy for improvement. Indeed, previous studies have found positive outcomes in changing HCP attitudes toward patients with SCD when videos of patients have been incorporated into the intervention.^17^ This may be related to intergroup contact theory,^35^ which states that positive contact between groups may reduce prejudice.^35^ Hearing directly from patients with SCD themselves may change HCPs’ attitudes toward these patients and increase willingness to learn new skills to address potential bias. Therefore, this suggestion may facilitate the uptake of the IPT skills from HCPs.

Despite criticisms of single-session implicit bias interventions, our focus group participants actually recommended keeping the intervention to one session, citing logistical barriers for HCPs that may prevent engagement in a multipart training. Other single-session interventions have demonstrated positive outcomes and stability over time compared to those with multiple sessions.^36^ Difficulty blocking time to schedule their assigned training session was the largest factor affecting attrition in our study. In particular, inpatient nurses had the greatest scheduling challenges, and many in the intervention condition indicated they were only able to do so because of managerial support. To address such logistical barriers, focus group participants proposed including the IPT intervention as part of their annual mandatory trainings. Findings also emphasize the importance of broader institutional support to promote staff participation in completing such interventions.

We were surprised by our findings that HCP pain treatment decision making did not differ between groups postintervention. In fact, all participants rated the preferred treatment plans higher than the less-preferred plans for all four virtual patient vignettes pretreatment. Though this finding contradicts previous research in adult SCD HCPs,^37,38^ the literature also points to possible explanations. Emergency department care for *pediatric* SCD pain is highly structured, with clear protocols and pathways that may prevent provider bias from impacting clinical decisions.^39^ The fact that nurses indicated somewhat lower agreement with preferred treatment plans than did physicians and advanced practice providers may indicate physicians/advanced practice providers have enhanced familiarity with recommended practices. Another consideration for why clinical decision making did not differ between groups postintervention is the study setting and expertise of our participants in pediatric SCD, given that we are the only comprehensive pediatric SCD program in the state. Previous research has found that compared to emergency department HCPs, hematology/oncology HCPs displayed greater knowledge of national published guidelines and best practices in SCD care.^40^

It was notable that, despite practicing in a highly diverse urban environment, HCPs in this study still demonstrated notable pro-White bias in their judgments of faces, both adult and child. Furthermore, the IPT intervention did not appear to impact these implicit judgments. Reductions in implicit biases do not reliably transfer to less biased treatment decisions.^41^ However, clinical care—in general and for pediatric SCD specifically—involves more than just HCP treatment decisions.^42^ As such, it is important to consider how bias manifests in these other domains. For example, microaggressions can arise in HCPs’ verbal and nonverbal communication with patients and caregivers.^43^ Such interpersonal manifestations of bias have been extensively documented in pediatric SCD care, where youth and their caregivers report racial bias and stigmatization from HCPs^9 – 12^ that fall outside the domain of “treatment decision making.” The clinical impact of such biases is indicated by studies showing that positive perceptions of HCP communication are linked to better outcomes for patients with SCD, including decreased admissions for pain and increased hydroxyurea adherence.^13^

Several important limitations to this study should be noted. First, our findings may not generalize to HCPs from other institutions, given our small sample of predominantly White, cisgender female HCPs recruited from one pediatric institution with a comprehensive pediatric SCD program. Second, we did not control for HCPs’ receipt of previous implicit bias training. However, we did use mild deception/incomplete disclosure at the beginning of the study to minimize the impact of social desirability and cognitive biases. Third, the use of the Race IAT has been criticized as a bias metric,^44^ though few alternative validated measures of implicit bias exist. Despite these limitations, this study is a step toward advancing the development of HCP-focused interventions to improve pediatric SCD pain care.

In conclusion, this mixed methods pilot study is the first to evaluate an IPT intervention to reduce bias and improve pain treatment clinical decision making among pediatric SCD HCPs. Findings indicate that although the intervention did not appear to impact their implicit or explicit biases or pain treatment decisions, HCPs engaged in the intervention and provided positive feedback on its format, content, and impact. Suggestions for intervention refinement include shortening its overall length, increasing time for IPT skills practice, and incorporating greater involvement from patients. These enhancements are likely to increase the intervention’s clinical utility. Future research is needed to assess patient and caregiver perspectives of the IPT intervention and to clarify the impact of the intervention on different manifestations of HCP bias (e.g., communication), as well as its ability to yield improvements in health outcomes for youth with SCD.

## Supplementary Material

Supplemental_File_Codebook.docx

Supplemental_File_FG _Questions.docx

241314229R2_Main_Document.docx
