# Supplementary material for: Initial evaluation of an intervention to address provider implicit bias in pediatric sickle cell disease pain care: A mixed methods pilot study
Source: Can J Pain. 2025 May 9;8(2):2486819. doi: 10.1080/24740527.2025.2486819 (PMC12068330; doi:10.1080/24740527.2025.2486819)
Supplement: Supplemental_File_Codebook.docx [file UCJP_A_2486819_SM2374.docx]

**Figure 4.** *Codebook for Thematic Analysis of Focus Group Transcripts*

| **Acceptability (quality of being satisfactory, agreed to or approved of; ability to meet needs)** | | | |
| --- | --- | --- | --- |
| content | applicable to their job | structure | good flow |
|  | definitions were helpful |  | good order of topics |
|  | easy to understand/clear |  | well organized |
|  | good reminder/confirmatory | suggestions | include more actionable items |
|  | good/acceptable |  | offer during meetings/retreats/residency educational sessions |
|  | interactive |  | efficient with time |
|  | not enough/too basic |  | give materials/questions in advance of training |
|  | not new |  | offer during grand rounds |
|  | questions were thought-provoking |  | have SCD patients join training session |
|  | Redundant |  | include discussion of pain |
| group size | Good |  | offer as a lunch session |
|  | too big* |  | more interactive |
|  | too small |  | offer during orientation/onboarding |
| include topics other than race | class/socio-economic status |  | include more practice |
|  | gender identity |  | use recorded session |
|  | other disparities |  | offer refreshers |
| "Poll Everywhere" feature | did not like this feature |  | offer during annual validations |
|  | like this feature | time | just right |
| presenter | good speaker |  | need more time at end for discussion |
|  | Great |  | too long |
|  | too slow |  | too short |
| similarity to other training | different/unique from other trainings | videos | Helpful |
|  | focused on SCD |  | impactful |
|  | too similar to previous trainings |  | not enough |
|  |  |  | too many |
| **Feasibility (degree of being possible, practical, or easily done)** | | | |
| barriers | anxious about discussion on race | key take-aways | importance of pausing |
|  | lack of interest |  | individual mindset |
|  | need for departmental/organizational support |  | perspective-taking |
|  | no change |  | recognizing own biases* |
|  | time requirement is barrier |  | see patients as people/look for similarities with patients |
| feasible | can be implemented |  | striking |
|  | can fit into existing meetings |  | stigma* |
|  | will be difficult to schedule |  | working with challenging patients |
| in-person/  virtual | in-person | frequency requirement | less than yearly |
|  | mix/combo |  | more than yearly |
|  | Virtual |  | tailored to role |
| should be required? | not for all providers on a specific disease |  | yearly |
|  | Yes |  |  |

* code included, but not selected
