# Supplementary material for: Initial evaluation of an intervention to address provider implicit bias in pediatric sickle cell disease pain care: A mixed methods pilot study
Source: Can J Pain. 2025 May 9;8(2):2486819. doi: 10.1080/24740527.2025.2486819 (PMC12068330; doi:10.1080/24740527.2025.2486819)
Supplement: Supplemental_File_FG _Questions.docx [file UCJP_A_2486819_SM2373.docx]

**SCD Provider Implicit Bias Study:**

**Provider Focus Group Questions**

Feasibility of the Intervention

- How feasible would it be for you to receive this intervention?
- What did you think about the organization of the material presented and the teaching methods used during the intervention?
- What did you like/dislike about the structure and format of the intervention?
- What did you think about the virtual format?
- Was the time allotted too long or short?
- Would the intervention session fit into your department/clinic workflow? Why or why not?
- Do you think this intervention should be required for all providers? Why or why not? If you think it should be required, when and how often do you think providers should receive it? Why?
- What (logistical) barriers might interfere with your participation in the intervention? Why? How, if at all, could these barriers be addressed?

Acceptability and Impact of the Intervention

- What did you think about the intervention?
- What stood out to you about the information provided?
- Do you think the intervention was relevant to your clinical work with sickle cell patients? Why or why not?
- Do you think it was helpful to attend the intervention? Why or why not?
- What, if any, were some of the (cognitive/emotional) challenges you experienced remaining engaged or understanding the material? How, if at all, were these challenges similar to or different from previous clinical trainings you may have attended?

Prompts: How could that have been better? What suggestions do you have to improve this?
