# Supplementary material for: Initial evaluation of an intervention to address provider implicit bias in pediatric sickle cell disease pain care: A mixed methods pilot study
Source: Can J Pain. 2025 May 9;8(2):2486819. doi: 10.1080/24740527.2025.2486819 (PMC12068330; doi:10.1080/24740527.2025.2486819)
Supplement: 241314229R2_Main_Document.docx [file UCJP_A_2486819_SM2372.docx]

**Initial Evaluation of an Intervention to Address Provider Implicit Bias in**

**Pediatric Sickle Cell Disease Pain Care: A Mixed Methods Pilot Study**

Siddika S. Mulchan, Psy.D.^1,2^, Christopher B. Theriault, M.A.^1^, Susan DiVietro, Ph.D.^1,2,3^, Mark D. Litt, Ph.D.^4^, Emily O. Wakefield, Psy.D.^1,2^, Javeed Sukhera, M.D., Ph.D.^5^, Paula Tanabe, Ph.D.^6^, Hannah R. Thomas, M.S.^7^, Melissa Santos, Ph.D.^1,2^, William T. Zempsky, M.D., M.P.H.^1,2^, Donna Boruchov, M.D., ^1,2^ Adam T. Hirsh, Ph.D.^8^

^1^Connecticut Children’s, ^2^Department of Pediatrics, University of Connecticut School of Medicine, ^3^Injury Prevention Center, University of Connecticut, ^4^Department of Behavioral Sciences, UConn Health, ^5^Department of Psychiatry, Hartford Hospital, ^6^Duke University School of Nursing and Medicine, Durham, NC, ^7^Department of Psychological Sciences, University of Connecticut, ^8^Indiana University Indianapolis

**Corresponding Author:** Siddika S. Mulchan https://orcid.org/0000-0002-8380-3735

Center for Cancer and Blood Disorders, Connecticut Children’s, 282 Washington Street, Suite 5A, Hartford, CT, 06106. Phone: (860) 545-8122, Fax: (860) 545-9622, Email: [ssmulchan@gmail.com](mailto:ssmulchan@gmail.com)

**Funding:** This work was supported by the Hartford Foundation for Public Giving.

**Abstract**

**Background:** Health care provider (HCP) implicit bias can impact health outcomes for youth with sickle cell disease (SCD). **Aims**: Evaluate the feasibility, acceptability, and preliminary impact of an Individuation and Perspective Taking (IPT) intervention to decrease implicit bias and improve pain treatment clinical decision-making in pediatric SCD HCPs. **Methods:** This mixed methods pilot randomly assigned HCPs (*N* = 36) to an intervention (*n* = 17) or control condition (*n* = 19). Implicit and explicit bias measures were administered pretreatment and 3-months post-intervention. Differences were analyzed using repeated measures analyses of variance. HCP ratings of virtual patient vignettes depicting Black and white youth with SCD or cancer pain were used to assess differential clinical decision-making based on race and diagnosis and analyzed using hierarchical linear mixed model analysis. Focus groups with intervention participants were analyzed using thematic analysis. **Results:** No significant differences in scores on bias measures across time, condition, or the condition X time interaction were found (all *p* < .05). Significant differences in HCP ratings were found between types of HCPs (*p* < 0.001), but no effects were attributable to condition, time, virtual patient race or diagnosis. Ten themes were extracted regarding the intervention’s format, structure, and content. **Conclusions:** This study is the first to evaluate an IPT intervention in pediatric SCD HCPs. HCPs deemed the intervention feasible, acceptable, and impactful, and suggested areas for improvement. Future research should refine the intervention to incorporate greater patient involvement and skills practice to improve health outcomes for this underserved population.

*Key words: sickle cell disease, pediatrics, implicit bias, mixed methods*

**Introduction**

Sickle cell disease (SCD) is a genetic blood disorder that primarily affects individuals of sub-Saharan African descent^1^. It is the most common genetic disorder in the United States, affecting over 100,000 Americans^2^. Approximately 2,000 babies are born with SCD in the United States each year^3^. Many patients experience both acute and chronic pain that can begin in childhood, especially during the adolescent and young adult developmental period. Youth with SCD are at high risk for poor health outcomes due to a combination of individual and systemic factors affecting access to quality care, adherence to medical regimens, and biases among health care providers (HCP; physicians, physician assistants, nurse practitioners, and nurses)^4,5^.

There have been increased calls to address implicit bias from HCPs in SCD care, as numerous studies have documented the negative impact of bias on health outcomes^4,6^. Implicit bias are negative attitudes that occur outside of conscious awareness, whereas explicit biases are conscious and deliberate^4^. In pediatric SCD, HCP bias has been linked to pain undertreatment, low rates of hydroxyurea prescription and referrals for transcranial Doppler screenings, and gaps in treatment during the transition from pediatric to adult care^4,7,8^, when SCD patients’ risk of mortality increases more than twofold^5^. Youth with SCD and their caregivers report experiencing racial bias and discrimination from HCPs^9–12^ in the emergency department and outpatient setting, which can harm the patient-provider alliance and exacerbate negative health outcomes^13^. HCPs who treat SCD often also provide care to individuals with cancer. Interestingly, a qualitative study with Black adults with SCD or cancer found that ^14^while both groups reported experiencing delays in treatment and poor communication from HCPs, only those with SCD reported perceived discrimination, being accused of drug-seeking, and feeling unheard by their HCPs^14^.

Previous research has proposed strategies to mitigate implicit bias, such as individuation and perspective-taking. Individuation involves focusing on unique personal qualities of the patient rather than group-based characteristics, while perspective-taking refers to considering patient experiences from the patient’s point of view^15,16^. We reviewed the relevant literature and could not find any studies that reported having developed and/or tested an individuation and perspective-taking intervention to address HCP bias.

Previous provider-focused interventions have mostly focused on educational content targeting attitudes and beliefs about caring for patients with SCD^17,18^. One study tested the impact of an 8-minute video intervention compared to no intervention on the attitudes of nurses and house staff toward adults with SCD. The video consisted of a hematologist and three patients with SCD discussing challenges associated with SCD pain care. The results showed decreased negative attitudes and increased positive attitudes toward patients with SCD among the intervention condition compared to the no intervention control condition^17^. Another study explored the feasibility and acceptability of a Health Equity Extension for Community Healthcare Outcomes training. This program consisted of monthly, one-hour virtual sessions with didactic presentations and group-based discussion focused on racism and racial justice, as well as strategies to promote change at various levels (e.g., individual, institutional, etc.). Preliminary analyses indicated that the intervention showed promise in increasing HCP self-awareness about implicit bias and racism and in building social justice skills^18,19^. These studies, though encouraging, have not exclusively targeted pediatric SCD HCPs nor produced a validated intervention, and are limited in their ability to evaluate the impact of such interventions on HCP behavior, including clinical decision-making.

The primary objectives of this mixed methods pilot study were to explore the feasibility, acceptability, and preliminary impact of an Individuation and Perspective Taking (IPT) intervention intended to decrease implicit bias and improve the effectiveness of pain treatment clinical decision-making in pediatric SCD HCPs. Previous research has that proposed training based on IPT may mitigate bias^15,16^. We hypothesized that, compared to HCPs in a didactic information control condition, HCPs assigned to the IPT intervention would demonstrate: 1) lower levels of implicit racial bias as assessed by the Race Implicit Association Test (IAT^20^) and explicit bias as assessed by an explicit SCD bias measure^21^ and 2) more effective pain treatment clinical decision-making (i.e., consistent with published guidelines for care) as assessed by ratings on virtual patient vignettes. A secondary objective was to explore differences in pain treatment decisions for virtual patients with SCD vs. cancer pain, given that both diagnoses are treated by pediatric HCPs within the hematology/oncology subspecialty. Based on previous research^14^, we expected that HCPs would make more effective pain treatment clinical decisions for virtual patients with cancer. Finally, we conducted focus groups with HCPs in the intervention arm to elicit their perspectives on the feasibility, acceptability, and preliminary impact of the IPT intervention. We hypothesized that HCPs’ responses would largely support the feasibility and acceptability of the intervention.

**Methods**

This explanatory sequential mixed methods pilot study was conducted in a web-based format at a mid-sized, freestanding children’s hospital in the northeastern United States. Approval from the Connecticut Children’s Institutional Review Board (IRB) was obtained prior to all study procedures (CCMC IRB#: 21-036). The research design of the quantitative phase was a randomized pilot while the qualitative phase used a descriptive-interpretative design. Study goals were to understand complex phenomena (i.e., implicit and explicit bias) and measure change over time. The objective of the quantitative phase was influence of the intervention on HCP bias and pain treatment clinical decision-making, while exploration of the feasibility, acceptability, and overall impact of the intervention was the objective of the qualitative phase. Mixed methods were used for the purpose of participant enrichment^22^ to determine whether the intervention contributed to change (e.g., less bias) and how it impacted participants. IPT is intended to impact HCPs’ communication with patients to reduce biases. Given that this study was a pilot study, it was not registered in a clinical trails database.

***Participants***

A sequential-nested sampling design was used in this study. Participants were 36 physicians, advanced practice providers, and registered nurses who provide care to youth with SCD in the inpatient and/or outpatient (i.e., clinic-based) setting. Advanced practice providers were physician assistants and nurse practitioners. Participants consisted of primarily white (*n* = 33; 91.6%), cisgender females (*n* = 32; 88.9%) with a mean age of 38.6 years. Participants included physicians (*n* = 10; 27.8%), nurses, (*n* = 11; 30.6%), advanced practice providers (*n* = 8; 22.2%), and medical trainees (*n* = 7; 19.4%). On average, most participants (*n* = 21; 58.3%) reported having at least 6 years of experience caring for patients with SCD. See Table 1 for participant characteristics.

Participants were recruited from the Hematology/Oncology and Emergency Medicine Departments, and from inpatient medical/surgical units. Recruitment occurred by contacting department heads and administrative staff to share study information with eligible participants via team huddles, email, and IRB-approved flyers posted in staff breakrooms. Inclusion criteria were: 1) older than 18 years of age, 2) provided direct patient care for at least 10 hours per week during the study period, 3) licensed or certified health care professional or medical trainee, 4) English fluency and ability to provide informed consent.

A total of 52 participants initially expressed interest in study participation, completed pretreatment measures, and were randomly assigned to the intervention or control condition. However, 15 of these participants (29%) were either unable to be scheduled for the IPT intervention or control session due to lack of availability (*n* = 6), were lost to follow-up (*n* = 4), withdrew from the study (*n* = 4), or were no longer employed at the institution (*n* = 1). Participants were emailed up to three times to schedule their session. Those who did not respond were considered lost to follow-up. Additionally, one participant was lost to follow-up at 3-months post-intervention (never responded to emailed post-intervention survey links), resulting in a total *N* = 36 (*n* = 17 intervention participants, *n* = 19 control participants) for all analyses. See Figure 1 for a diagram of participant recruitment, retention, and attrition. Five focus groups (3-4 participants in each) were completed with all 17 intervention participants.

***Measures***

**Demographic Questionnaire.** Participants completed a demographic questionnaire pretreatment that assessed their age, race/ethnicity, gender identity, role, primary work location, years of experience in their current profession, and years of experience treating patients with SCD.

**Race IAT.** Participants’ implicit attitudes about race were assessed with the Race IAT^20^. The Race IAT has been widely used as a measure of implicit racial bias^20,23,24^, including among HCPs^25^. On the Race IAT, participants categorize – as quickly as possible without making errors – facial images as depicting a Black or white person and evaluative words as good or bad (e.g., “pleasure” is a good word and “awful” is a bad word). Separate computer keys are used to make these categorical judgments. In one trial, participants were instructed to use the same key to indicate a white face or good word and a different key to indicate a Black face or bad word. In a second (reverse) trial, participants were instructed to use the same key to indicate a white face or bad word and a different key to indicate a Black face or good word. Faster responses to the white/good + Black/bad pairings than to the white/bad + Black/good pairings indicated stronger positive bias associated with white over Black people^26^, and vice versa. Participants received a *D*-score ranging from -2 to +2, with positive values indicating more positive bias associated with white people and negative values indicating more positive bias associated with Black people^26^. A common rule of thumb is that values of 0.15–0.34 indicate slight positive bias, 0.35–0.64 moderate positive bias, and ≥0.65 strong positive bias^26^.

Two Race IATs were coded into the Qualtrics survey for the purpose of this study, one with adult faces and the other with child faces. At each time point (pretreatment and 3-months follow-up), participants completed the Race IAT twice, once with the adult faces and once with the child faces, which yielded two separate scores. The order in which the two versions were presented was randomized to reduce testing bias.

**Virtual Patient Vignettes.** Virtual patient vignettes (see Figures 2 and 3) were developed to assess HCP pain treatment clinical decision-making^27^. Virtual patient technology has been used in previous studies to assess the impact of patient demographic variables, including race/ethnicity, on pain assessment, clinical decision-making, and treatment disparities^28–30^. Virtual patient vignettes for this study, including treatment questions and response options, were systematically modeled after those used in previous research^21^, and pilot tested in pediatric hospitalists and medical trainees^27^.

Participants were shown four unique, animated virtual patient vignettes (2 Black male adolescents with SCD, and 1 Black and 1 white male adolescent each with acute lymphocytic leukemia). Each video was 60 seconds long and showed virtual patients exhibiting pain behavior in an emergency department hospital room. The animations were accompanied by text descriptions of the patient and presenting problem, along with physiological data (e.g., temperature, heart rate).

For each vignette, providers were asked to rate their agreement with each of two pairs of treatment plans, one pair pertaining to the initial stage of the encounter, and another pair of treatment plans pertaining to treatment once the patient had been evaluated. For each pair of plans at each stage, one of the pair represented a preferred treatment plan (i.e., one that was consistent with published guidelines for care or “best practices”) while the other plan deviated from guidelines. Providers rated each plan for each stage of the encounter on a scale from 1= “strongly disagree with this plan” to 5= “strongly agree with this plan.” Thus, each provider had four scores for each of the four vignettes. A “best practice score” for each vignette was calculated by taking the mean of the scores on the two preferred treatment plans, with higher scores indicating stronger agreement with treatment guidelines for care.

For example, when presented with a virtual patient with SCD (“James”; see Figure 2), at the initial stage of the encounter, physicians rated their level of agreement with: 1) Start intravenous morphine according to patient’s pain plan and reassess in 30 min, or 2) Start oral morphine according to patient’s pain plan since he can’t remember when he took his last dose and reassess in 30 min. After rating these decisions, physicians were presented with additional written information regarding the virtual patient’s status after having initial treatment (“After 3 doses of pain medication, James rates his pain as 10/10 and requests to be admitted”) and a second pair of treatment plans was presented, to which they indicated their agreement with: 3) Admit to the floor and start on morphine patient-controlled analgesia pump, or 4) Admit to the floor and start on intravenous morphine every 3 hours.

Nurses were presented with a different set of treatment plans for each vignette. When presented with the same virtual patient with SCD (“James”), nurses first rated their agreement with the following treatment plans: 1) Share patient’s request [for intravenous pain medication] with the attending provider and advocate for intravenous morphine, or 2) Start oral morphine according to patient’s pain plan since he can’t remember when he took his last dose and reassess in 30 minutes. Additional written information was presented to nurses after the virtual patient had received initial treatment (“After 3 doses of pain medication, James rates his pain as 10/10 and requests to be admitted. You observe him smiling and laughing loudly while talking on the phone as you prepare for his admission”). Nurses then rated their agreement with: 3) Inform the attending provider that the patient wants to be admitted and report your observation to inform the plan of care, or 4) Inform the attending provider that the patient wants to be admitted.

**Explicit SCD Bias.** Explicit bias against youth with SCD was assessed by using a measure of explicit bias from a previous study^21^, which was based on other validated measures of explicit bias^31^. Participants rated their level of agreement on a 5-point Likert scale ranging from 1 = “strongly disagree” to 5 = “strongly agree” with three statements that reflect negative stereotypes of SCD patients: 1) Treating youth with SCD is more challenging than treating youth with other illnesses, 2) Youth with SCD seem to present with less urgent issues than youth with other illnesses, and 3) Youth with SCD are often less compliant than youth with other illnesses. The item scores were summed to create an explicit bias total score, ranging from 3 to 15, with higher scores indicating stronger agreement with SCD stereotypes.

***Procedures***

Prospective participants were emailed a unique link to study measures. Measures were administered at two time points (pretreatment and 3-months post-intervention) via Qualtrics, a web-based survey tool. Measures were administered at 3-months follow-up for the following reasons: 1) to reduce testing effects from completing the Race IATs, 2) to reduce participant burden, and 3) to determine retention of the IPT skills, given that few implicit bias interventions facilitate sustained change over time^32^. Participants provided informed consent electronically by selecting “yes” prior to beginning the survey. Our study procedures are at increased risk for socially desirable and biased responding from participants. To minimize this, we received IRB approval to use mild deception/incomplete disclosure during the consent process, such that the study was described as focusing on clinical decision-making rather than implicit bias. Measures were completed at participants’ convenience by a given deadline. After completing pretreatment measures, participants were given a study ID and randomly assigned to the intervention or control condition by C.B.T. Randomization was conducted with a random integer sequence generator (RANDOM.ORG) which generates randomness based on atmospheric noise^33^. Integers corresponded to participant study ID numbers and the sequence was produced as two, separate even columns for either the intervention or control group. Only C.B.T. had access to participants’ assignments; all other members of the research team were blinded to participant assignment.

Sessions were conducted in small groups and scheduled based on participants’ availability, which resulted in 5 intervention sessions (3-4 participants in each) and 6 control sessions (3-4 participants in each). There was one more control than intervention session due to differences in the overall group size (*n* = 20 control participants vs. *n* = 17 intervention participants). Intervention participants were also invited to participate in focus groups immediately post-intervention to explore the feasibility, acceptability, and overall impact of the intervention. Focus groups were conducted as soon as possible following participants’ completion of the intervention to facilitate recall and retention. To minimize social desirability bias, focus group participants were told that they would remain anonymous, their responses would be kept private and confidential, and they were not required to turn on their video cameras during focus groups sessions. Three-months post-intervention, all participants were emailed a link up to three times to complete posttest measures via Qualtrics. Following the completion of posttest measures, participants were emailed a form describing the use of deception/incomplete disclosure, alongside a short video of the PI (S.S.M.) explaining why this was used. Participants were compensated with a $25 VISA gift card for completing the pretreatment survey, a $35 VISA gift card for participating in the intervention, a $40 VISA gift card for participating in focus group sessions (if applicable), and a $50 VISA gift card for completing the 3-month follow-up survey.

**Intervention and Control Conditions.** The intervention group participated in a 90-minute, live virtual session conducted via Zoom using didactic and experiential learning that heavily focused on the use of two cognitive strategies that have been previously proposed to reduce bias among HCPs (individuation and perspective-taking^15^. The IPT intervention was developed in consultation with experts in implicit bias and SCD pain care and conducted by an expert in the field (not an author or member of the research team). The content consisted of a brief didactic presentation on the cycle of socialization, neurobiology of implicit bias, and history of medical racism relevant to the SCD population, followed by an in-depth explanation of the IPT cognitive strategies and practice using these skills with publicly available SCD patient videos (https://www.youtube.com/watch?v=yCOvkOCaaSk). The control condition was exposed to a 60-minute, pre-recorded virtual session about best practices in pediatric SCD pain management delivered by a medical expert in pediatric SCD pain (W.T.Z.).

**Focus Groups.** Intervention group participants were invited to participate in a 30-minute virtual focus group post-intervention via Zoom. When possible, focus groups were conducted immediately post-intervention to facilitate participant recall and retention. Five focus groups of 3-4 participants each were conducted by trained research personnel (C.B.T. and S.D.) using a semi-structured format and questioning route, which is the planned sequence of questions, beginning with broad questions and becoming increasingly focused on the construct(s) of interest. Focus group questions centered on the intervention’s feasibility (e.g., format, length, etc.), acceptability (e.g., relevance to participants’ work), and overall impact (e.g., interest in maintaining IPT skills and applying them to patient care), and elicited feedback on areas for improvement. Questions included: *Do you think the intervention was relevant to your clinical work with sickle cell patients? Why or why not?* (acceptability) and *What did you like/dislike about the structure and format of the intervention?* (feasibility). The average duration of focus groups was approximately 25 minutes. Each focus group was audio recorded, de-identified, and transcribed using a secure transcription service. Transcriptions were then reviewed for accuracy by other members of the research team prior to conducting a thematic analysis.

***Data Analysis***

**Quantitative.** The Statistical Package for Social Sciences (SPSS) Version 20 was used for all statistical analyses. Descriptive statistics were calculated for participant demographics. Frequency tables, along with the means and standard deviations, were calculated for the Race IAT scores with adult and child faces and responses to the explicit SCD bias measure at pretreatment and 3-months post-intervention. Means and standard deviations were also calculated separately for physician/advanced practice provider and nurse virtual patient vignette best practice ratings.

Race IAT scores for both adult and child faces were analyzed separately using mixed effect repeated measures analyses of variance (ANOVAs). Analyses were performed to examine the main effects of condition (control vs. intervention), time (pretreatment and 3-months post-intervention), and the condition x time interaction on Race IAT D scores.

As described above, for each virtual patient, providers made two agreement ratings (initial stage and following evaluation). The “best practice score” was calculated as the mean of the agreement ratings on the two preferred treatment plans. These best practice scores were analyzed using a hierarchical linear mixed model analysis. The scores for multiple patients (the two patients with SCD and the two with cancer) were nested within subject within Time. The analysis modeled the main effects of Condition (control vs. intervention), Time (pretreatment vs. 3 months follow-up), Provider Type (nurse vs. physician or advanced practice provider), the race of the patient (Black vs. white), and the diagnosis of the patient (SCD vs. cancer), plus the interactions of Condition X Time, Condition X Patient Race, Condition X Patient Diagnosis, and Condition X Time X Patient Race. We were thus able to determine if HCPs’ clinical decision-making differed by treatment, patient race, patient diagnosis, and by whether the HCP was a prescriber (physician or advanced practice provider) or a nurse, as well as whether any of these interacted with other status variables.

Finally, explicit bias scores were analyzed using repeated measures ANOVA. Bias scores were analyzed for differences attributable to Condition, Time, Provider Type, Condition X Time, Provider Type X Time, and Condition X Time X Provider Type.

**Qualitative.** An inductive thematic analysis^34^ of focus group transcriptions was conducted using HyperRESEARCH Version 4.5.4. HyperRESEARCH was selected for qualitative analyses due to its affordability, user-friendly interface, enhanced collaboration tools, extensive customer support, and authors’ previous experience using this software. Two study personnel (C.B.T. and S.D.) reviewed the focus group transcripts independently and created codebooks to identify key themes extracted from the focus group sessions. The personnel met to discuss and compare findings. Coding differences were resolved through consensus. A master codebook was then created to summarize key themes that were extracted from the data. Results were triangulated using a brief, 5-item intervention feedback form that evaluated participants’ expectations and perceived utility of the IPT intervention and whether they would recommend it to a friend. Participants were also asked to write down 3 things they learned from the intervention and what they would change to improve the intervention.

***Data Validation/Legitimation.***

**Quantitative.** Threats to internal validity were addressed by including a control group and randomly assigning participants to a condition. Threats to external validity were addressed by including clear inclusion/exclusion criteria, using a pre/post design to reduce testing effects, and using mild deception/incomplete disclosure to minimize social desirability bias.

**Qualitative.** Threats to data trustworthiness and credibility were addressed by triangulating themes from focus groups with the intervention feedback form. Participants’ responses were summarized at the end of each focus group to elicit feedback/corrections further supported data credibility.

**Results**

**Quantitative Data**

There were no missing data from survey and Race IAT responses for any participant between pretreatment and follow-up. Means and standard deviations for all outcome measures are presented by treatment condition (intervention vs. control) across the two time points (pretreatment and 3-months follow-up) in Table 2.

***Race IAT***

Categorical preference tendencies for white vs. Black faces are shown in Table 3. Participants demonstrated a clear pro-white bias for both adult and child faces pretreatment. For adult faces, 80.6% of our sample demonstrated a pro-white bias, with the largest percentage demonstrating a *strong* pro-white bias (36.1%), while only 16.7% demonstrated no bias. For child faces, 94.4% of participants demonstrated a pro-white bias, with the largest percentage (47.2%) demonstrating a *moderate* pro-white bias, and only 5.6% demonstrating no bias. At 3-months post-intervention, participants continued to show pro-white biases for both adult and child faces. For adult faces, 77.8% of our sample demonstrated a pro-white bias, with the largest percentage being a *moderate* pro-white bias (38.9%) and 16.7% showing no bias. For child faces, 80.1% of participants demonstrated a strong pro-white bias, with the largest percentage being a *strong* pro-white bias (30.6%) and 13.9% showing no bias.

Results of the repeated measures ANOVAs on IAT D scores indicated that there were no main effect for intervention condition or time on IAT scores of adult faces or child faces, nor was there a condition X time interaction (see Table 4).

***Virtual Patient Vignettes***

Pretreatment and 3-months post-intervention, all participants rated the preferred treatment plans higher than the alternative for each of the four virtual patients. Table 5 shows the results of the linear mixed model analysis of treatment plan agreement ratings, aggregated over all four virtual patients. As seen in the table, there were no effects on treatment plan ratings attributable to condition (intervention or control), time, virtual patient race or diagnosis. There were differences seen between types of providers, such that physicians and advanced practice providers tended to indicate more agreement with the preferred treatment plans than did the nurses (*M* for physicians and advanced practice providers = 3.94, *SD* = 0.95; *M* for Nurses = 3.19, *SD* = 0.89).

***SCD Explicit Bias Measure***

All participants demonstrated low levels of agreement with negative stereotypes about youth with SCD pretreatment (*M* = 6.86, *SD* = 1.94). Just under half (*n* = 16, 43.2%) agreed that treating sickle cell disease is more challenging than treating other illnesses. Only 1 (2.7%) agreed that sickle cell patients are less compliant than youth with other illnesses, and only 5 (13.5%) agreed with the statement that sickle cell patients present with less urgent issues than patients with other illnesses. At follow-up, scores remained similar (*M* = 7.33, *SD* = 2.11). Approximately half (*n* = 18; 48.6%) agreed that treating sickle cell disease is more challenging than treating other illnesses. Only 1 (2.8%) still agreed that sickle cell patients are less compliant than youth with other illnesses, and 8 (22.2%) agreed with the statement that sickle cell patients present with less urgent issues than patients with other illnesses.

Results of the repeated measures ANOVA indicated that the intervention and control group did not significantly differ in their scores on the SCD explicit bias measure, nor were there any effects on explicit bias attributable to Time or to Provider Type (see Table 6).

**Qualitative Data**

Analysis of transcripts of focus groups with intervention participants identified 10 themes related to the feasibility, acceptability, and impact of the intervention, as well as specific recommendations on strategies for improvement. Themes included: logistical barriers, virtual format, well-organized (feasibility), relevant/applicable to work, training requirement, similar to previous training (acceptability), patients’ lived experiences, skills practice and mindset, more practice with skills, and patient involvement (strategies for improvement; see Table 7).

***Feasibility***

Focus group participants agreed that the IPT intervention was well-organized and that the presenter was highly knowledgeable. One nurse shared, “I thought the virtual format worked well, and I think [she] was a really great speaker. I liked the way that she presented and incorporated us into the discussion” (Participant #28, hematology/oncology nurse, 6-10 years of experience). While there was general agreement that the intervention should be required for all clinical staff, many participants highlighted logistical barriers to offering this education to HCPs at all levels of care (e.g., inpatient, outpatient, etc.). They expressed concerns about its length, noting that 90 minutes could be difficult to fit into busy clinical schedules. One nurse indicated, “I think this is important work and, you know, any of the training that we do is certainly important. It's just a matter of carving out time or finding staffing to make sure that from a nursing standpoint, we can staff that for a while” (Participant #38, iInpatient nurse, <1 year of experience). The time requirement was the most consistent obstacle identified.

To address logistical issues, participants recommended scheduling the intervention to coincide with existing trainings/meetings, such as during employee orientation/onboarding, Grand Rounds, lunch sessions, and/or required annual education. They also emphasized the need for institutional support for the training. One medical trainee said, “It would need to be like an evening or put into one of our didactic slots . . . . it would need to fit into a space that is already reserved for some type of educational session” (Participant #48, pediatric resident).

Participants recommended that the intervention stay virtual, acknowledging that virtual trainings are easier for staff to attend, and thus may help overcome some of the logistical challenges. Some participants noted that this was a very personal training with potentially sensitive discussion, which could be even more effective in person. One nurse noted, “I really did like the small group. I was a little nervous because it kind of forced you to talk more, but also I felt more comfortable because I could see and I knew . . . who I was talking to” (Participant #28, hematology/oncology nurse, 6-10 years of experience). A few participants suggested a hybrid model but indicated that a hybrid approach may introduce still more logistical hurdles. One nurse observed, “The virtual [format] is nice because you can always get everyone in the same room at the same time” (Participant #20, hematology/oncology nurse, more than 15 years of experience).

***Acceptability***

Most focus group participants found the IPT intervention to be relevant and applicable to their job and a good reminder of prior trainings. A nurse reflected, “I see the importance and I think that this is very relevant” (Participant #38, inpatient nurse, <1 year of experience). While a few participants found the content redundant, or too similar to previous instruction, many articulated that the intervention was important and valuable to their work. Some noted that they had previously learned a lot about racism and discrimination, and highlighted the importance of demonstrating how implicit biases directly impact patient care, specifically for SCD patients. They also noted that the patient videos helped keep them engaged with the content. One medical trainee shared, “I thought that the smaller group was really helpful just because . . . . it's really intimidating to talk in front of a lot of people about things that you may be feeling that are more personal” (Participant #51, pediatric intern).

***Impact***

For many, the most impactful and helpful parts of the intervention were the videos of children and families talking about their experiences with SCD, which HCPs used to practice the IPT skills. The videos showed the real experiences of patients and caregivers, in their own words, and helped HCPs understand the clear value of seeing patients as individuals (individuation) and understanding the interactions from the patient’s perspective (perspective taking). For example, one nurse noted, “My favorite was the [video]. Hearing . . . . from the patients themselves, what they go through. We see the kids when they come into the hospital for crisis; we don't see them when they're doing well at home. So that was really helpful for me” (Hematology/oncology nurse, 6-10 years of experience). Others noted that, until recently, HCPs referred to sickle cell patients as “sicklers,” thus failing to see them as individuals and reducing them to their disease. The videos helped reinforce that different patients deal with pain differently, and that HCPs often see patients on their worst days. Focus group participants highlighted the value of both learning and practicing the IPT skills. Several indicated that there was not enough time at the end of the training to practice these skills. They noted that too much time was spent laying out the issue and providing background about implicit bias; they would have preferred to have had more time to practice the IPT skills.

**IPT Skills.** Many participants reflected on the particular skills associated with the IPT intervention and saw clear pathways for how these skills could inform their clinical work. Some identified how this training could improve their effectiveness as HCPs. One medical trainee observed: “It's always good to be reminded, to take a step back and think about what everyone else is going through and kind of what might be contributing to why [the patient’s] acting a certain way . . . .” (Participant #45, pediatric resident). Others articulated how to apply the lessons and skills to their current work, laying out easy steps that can help reinforce the goals of individuation and perspective-taking. One nurse practitioner reflected:

“Before you go into the room, trying to remember something that you know about them that's totally separate from why they're in the hospital. I think trying to connect – I think that's such an important tool and we know that patients do better when they feel connected and trust their providers. And we do a better job of taking care of patients when we feel connected to our patients and we care about them. So really trying to remember that stuff. It's not just about knowing that they are an individual but connecting to them” (Participant #1, hematology/oncology nurse practitioner, 6-10 years of experience).

Many participants discussed the importance of slowing down and taking a breath before interacting with their patients, taking a moment to acknowledge the pain and frustration that their patients and families are experiencing, and also using that time to remind themselves that they are real people, not a diagnosis.

***Suggested Improvements***

Focus group participants had specific suggestions for improving the IPT intervention. One suggestion was to include SCD patients in the intervention to enhance its impact by hearing directly from patients. One HCP suggested, “Like I said, I thought the cases were helpful, and seeing the kids’ videos were helpful. It’d be really impactful if you had an actual sickle cell patient to talk to us live via Zoom, for like five minutes” (Participant #18, emergency department physician, 10-15 years of experience). A nurse practitioner indicated, “ . . . . it would have also been helpful to hear the patient perspective of how providers can be helpful in their communication” (Participant #9, hematology/oncology nurse practitioner, more than 15 years of experience).

Participants also suggested incorporating tips on how to ask different kinds of questions into their clinical encounters, beyond the standard medical questions or questions about their pain, thus building on the goals of individuation, or seeing each patient as an individual. The vast majority of participants recommended shortening the didactic portion of the presentation in favor of more time to practice skills. Some even suggested practice changes as a result of the intervention, such as beginning each day and/or each team meeting with a short video or story of a patient’s perspective to get in the right mindset for the day ahead.

**Data Integration and Triangulation**

Quantitative and qualitative data were integrated using a triangulation strategy, which combines multiple perspectives for a comprehensive understanding of the research question. The qualitative and quantitative results were compiled and integrated to draw overall conclusions (see Table 8). First, we compared study surveys with focus group transcripts from intervention participants to identify patterns of convergence and divergence, which allowed us to obtain a more detailed and holistic evaluation of the IPT intervention. Results of the intervention feedback form corresponded to themes related to the intervention’s feasibility, acceptability, and overall impact. Specifically, both the feedback form and the focus group results emphasized the importance of addressing bias in providing care, and the relevance of the intervention. Additionally, suggested areas for improvement from both the intervention feedback form and focus group themes included incorporating real patient stories. While we did not find any significant differences pre- and posttreatment on the Race IAT, virtual patient vignettes, or SCD explicit bias measure as a function of the intervention, the combined quantitative findings led to inferences that were complementary and reinforcing to the qualitative findings. HCPs exhibited mild to moderate levels of pro-white bias, supporting the importance of bias in care provision, but clinical judgments were not affected by race or diagnosis of the patient. The intervention was well-received, but did not impact implicit bias or clinical decision-making. Thus, while perceived as relevant, the intervention was subject to improvement, both in form (e.g., duration) and content (e.g., inclusion of more real-life patient stories).

**Discussion**

This mixed methods pilot study is the first to evaluate a theoretically- and empirically-informed intervention to foster individuation and perspective-taking among pediatric HCPs caring for youth with SCD. In this initial evaluation of the intervention we compared HCPs randomized to one of two conditions (intervention vs. control) on measures of implicit (Race IAT) and explicit (SCD Explicit Bias measures) racial bias and HCP pain treatment decisions for virtual patient vignettes at two time points (pretreatment and 3-months follow-up). While the intervention and control conditions did not significantly differ in implicit or explicit bias or pain treatment decision-making, these findings are preliminary. Enhancements to the IPT intervention may make it more efficacious in future studies. HCPs who participated in the intervention nevertheless found it valuable, as evidenced by focus group findings indicating that the IPT intervention was deemed to be feasible, acceptable, and impactful. Focus group participants also had several suggestions for improving the intervention content and format, which could ultimately have implications for addressing provider bias and pain treatment decision-making.

Our sample of HCPs who received the intervention provided positive feedback overall on its format, content, and impact. They reported some overlap with previous implicit bias training but indicated that the focus on learning the IPT skills was novel and highly valued. As such, they recommended shortening the didactic portion of the intervention and allowing more time for IPT skills practice with SCD patient stories. Moreover, the inclusion of patient stories to practice these skills was a unique aspect of the IPT intervention and participants suggested greater patient involvement as one strategy for improvement. Indeed, previous studies have found positive outcomes in changing HCP attitudes toward patients with SCD when videos of patients have been incorporated into the intervention^17^. This may be related to intergroup contact theory^35^, which states that positive contact between groups may reduce prejudice^35^. Hearing directly from SCD patients themselves may change HCPs’ attitudes toward these patients and increase willingness to learn new skills to address potential bias. Therefore, this suggestion may facilitate the uptake of the IPT skills from HCPs.

Despite criticisms of single-session implicit bias interventions, our focus group participants actually recommended keeping the intervention to one session, citing logistical barriers for HCPs that may prevent engagement in a multi-part training. Other single-session interventions have demonstrated positive outcomes and stability over time compared to those with multiple sessions^36^. Difficulty blocking time to schedule their assigned training session was the largest factor affecting attrition in our study. In particular, inpatient nurses had the greatest scheduling challenges and many in the intervention condition indicated they were only able to do so because of managerial support. To address such logistical barriers, focus group participants proposed to include the IPT intervention as part of their annual mandatory trainings. Findings also emphasize the importance of broader institutional support to promote staff participation in completing such interventions.

We were surprised by our findings that HCP pain treatment decision-making did not differ between groups post-intervention. In fact, all participants rated the preferred treatment plans higher than the less-preferred plans for all four virtual patient vignettes pretreatment. While this finding contradicts previous research in adult SCD HCPs ^37,38^, the literature also points to possible explanations. Emergency department care for *pediatric* SCD pain is highly structured, with clear protocols and pathways that may prevent provider bias from impacting clinical decisions^39^. The fact that nurses indicated somewhat lower agreement with preferred treatment plans than did physicians and advanced practice providers may indicate physicians/advanced practice providers have enhanced familiarity with recommended practices. Another consideration that clinical decision-making did not differ between group post-intervention is the study setting and expertise of our participants in pediatric SCD, given that we are the only comprehensive pediatric SCD program in the state. Previous research has found that compared to emergency department HCPs, hematology/oncology HCPs displayed greater knowledge of national published guidelines and best practices in SCD care^40^.

It was notable that, despite practicing in a highly diverse urban environment, HCPs in this study still demonstrated notable pro-white bias in their judgements of faces, both adult and child. Furthermore, the IPT intervention did not appear to impact these implicit judgments. Reductions in implicit biases do not reliably transfer to less biased treatment decisions^41^ However, clinical care – in general and for pediatric SCD specifically – involves more than just HCP treatment decisions^42^. As such, it is important to consider how bias manifests in these other domains. For example, microaggressions can arise in HCPs’ verbal and nonverbal communication with patients and caregivers^43^. Such interpersonal manifestations of bias have been extensively documented in pediatric SCD care, where youth and their caregivers report racial bias and stigmatization from HCPs^9–12^ that fall outside the domain of “treatment decision-making.” The clinical impact of such biases are indicated by studies showing that positive perceptions of HCP communication are linked to better SCD patient outcomes, including decreased admissions for pain and increased hydroxyurea adherence^13^.

Several important limitations to this study should be noted. First, our findings may not generalize to HCPs from other institutions, given our small sample of predominantly white, cisgender female HCPs recruited from one pediatric institution with a comprehensive pediatric SCD program. Second, we did not control for HCPs’ receipt of previous implicit bias training. However, we did use mild deception/incomplete disclosure at the beginning of the study to minimize the impact of social desirability and cognitive biases. Third, the use of the Race IAT has been criticized as a bias metric ^44^, though few, alternative validated measures of implicit bias exist. Despite these limitations, this study is a step toward advancing the development of HCP-focused interventions to improve pediatric SCD pain care.

In conclusion, this mixed methods pilot study is the first to evaluate an IPT intervention to reduce bias and improve pain treatment clinical decision-making among pediatric SCD HCPs. Findings indicate that, although the intervention did not appear to impact their implicit or explicit biases or pain treatment decisions, HCPs engaged in the intervention and provided positive feedback on its format, content, and impact. Suggestions for intervention refinement include shortening its overall length, increasing time for IPT skills practice, and incorporating greater involvement from patients. These enhancements are likely to increase the intervention’s clinical utility. Future research is needed to assess patient and caregiver perspectives of the IPT intervention and to clarify the impact of the intervention on different manifestations of HCP bias (e.g., communication), as well as its ability to yield improvements in health outcomes for youth with SCD.

**Disclosure of Interest**

Financial interests: The authors have no relevant financial interests to disclose. Non-financial interest: William T. Zempsky, M.D., M.P.H. is a consultant for OmmioHealth and Editas, and a member of the Data and Safety Monitoring Board for Lundbeck, and Paula Tanabe, Ph.D., is a consultant for CSL Behring, Inc.

**References**

1. Kato GJ, Piel FB, Reid CD, et al. Sickle cell disease. *Nat Rev Dis Primers*. 2018;4(1):18010. doi:10.1038/nrdp.2018.10

2. Kavanagh PL, Fasipe TA, Wun T. Sickle Cell Disease. *JAMA*. 2022;328(1):57. doi:10.1001/jama.2022.10233

3. Sickle Cell Disease Association of America. FAQ Sickle Cell Disease.

4. Reich J, Cantrell MA, Smeltzer SC. An Integrative Review: The Evolution of Provider Knowledge, Attitudes, Perceptions and Perceived Barriers to Caring for Patients with Sickle Cell Disease 1970–Now. *Journal of Pediatric Hematology/Oncology Nursing*. 2023;40(1):43-64. doi:10.1177/27527530221090179

5. Lee L, Smith-Whitley K, Banks S, Puckrein G. Reducing Health Care Disparities in Sickle Cell Disease: A Review. *Public Health Reports*. 2019;134(6):599-607. doi:10.1177/0033354919881438

6. Hsu LL, Hooper WC, Schieve LA. Prioritizing Sickle Cell Disease. *Pediatrics*. 2022;150(6). doi:10.1542/peds.2022-059491

7. Cabana MD, Kanter J, Marsh AM, et al. Barriers to Pediatric Sickle Cell Disease Guideline Recommendations. *Glob Pediatr Health*. 2019;6:2333794X1984702. doi:10.1177/2333794X19847026

8. Schieve LA, Simmons GM, Payne AB, et al. *Vital Signs:* Use of Recommended Health Care Measures to Prevent Selected Complications of Sickle Cell Anemia in Children and Adolescents — Selected U.S. States, 2019. *MMWR Morb Mortal Wkly Rep*. 2022;71(39):1241-1246. doi:10.15585/mmwr.mm7139e1

9. Durgam N, Brion T, Lewis HB, et al. Patient and Caregiver Perspectives on Care-Seeking During a Vaso-Occlusive Crisis in Sickle Cell Disease: Results from Qualitative Interviews in Canada. *Patient Prefer Adherence*. 2023;Volume 17:41-49. doi:10.2147/PPA.S377924

10. Blakey AO, Lavarin C, Brochier A, et al. Effects of Experienced Discrimination in Pediatric Sickle Cell Disease: Caregiver and Provider Perspectives. *J Racial Ethn Health Disparities*. 2023;10(6):3095-3106. doi:10.1007/s40615-022-01483-4

11. Hood AM, Crosby LE, Hanson E, et al. The influence of perceived racial bias and health-related stigma on quality of life among children with sickle cell disease. *Ethn Health*. 2022;27(4):833-846. doi:10.1080/13557858.2020.1817340

12. Wakefield EO, Pantaleao A, Popp JM, et al. Describing Perceived Racial Bias Among Youth With Sickle Cell Disease. *J Pediatr Psychol*. 2018;43(7):779-788. doi:10.1093/jpepsy/jsy015

13. Cronin RM, Yang M, Hankins JS, et al. Association between hospital admissions and healthcare provider communication for individuals with sickle cell disease. *Hematology*. 2020;25(1):229-240. doi:10.1080/16078454.2020.1780737

14. Dyal BW, Abudawood K, Schoppee TM, et al. Reflections of Healthcare Experiences of African Americans With Sickle Cell Disease or Cancer: A Qualitative Study. *Cancer Nurs*. 2021;44(1):E53-E61. doi:10.1097/NCC.0000000000000750

15. Burgess D, van Ryn M, Dovidio J, Saha S. Reducing Racial Bias Among Health Care Providers: Lessons from Social-Cognitive Psychology. *J Gen Intern Med*. 2007;22(6):882-887. doi:10.1007/s11606-007-0160-1

16. Chapman EN, Kaatz A, Carnes M. Physicians and implicit bias: How doctors may unwittingly perpetuate health care disparities. *J Gen Intern Med*. 2013;28(11):1504-1510. doi:10.1007/s11606-013-2441-1

17. Haywood C, Lanzkron S, Hughes MT, et al. A Video-Intervention to Improve Clinician Attitudes Toward Patients with Sickle Cell Disease: The Results of a Randomized Experiment. *J Gen Intern Med*. 2011;26(5):518-523. doi:10.1007/s11606-010-1605-5

18. Shook LM, Crosby LE, Whitten-Shurney W, et al. A Health Equity Echo for Providers of Children and Adults with Sickle Cell Disease. *Blood*. 2022;140(Supplement 1):13141-13142. doi:10.1182/blood-2022-168716

19. Shook LM, Crosby LE, Farrell CB, Nelson SC. A health equity ECHO for clinicians of individuals with SCD. *Journal of Sickle Cell Disease*. 2024;1(1). doi:10.1093/jscdis/yoae005

20. Greenwald AG, Lai CK. Implicit Social Cognition. *Annu Rev Psychol*. 2020;71(1):419-445. doi:10.1146/annurev-psych-010419-050837

21. Puumala SE, Burgess KM, Kharbanda AB, et al. The Role of Bias by Emergency Department Providers in Care for American Indian Children. *Med Care*. 2016;54(6):562-569. doi:10.1097/MLR.0000000000000533

22. Leech NL, Onwuegbuzie AJ. Guidelines for Conducting and Reporting Mixed Research in the Field of Counseling and Beyond. *Journal of Counseling & Development*. 2010;88(1):61-69. doi:10.1002/j.1556-6678.2010.tb00151.x

23. Bar-Anan Y, Nosek BA. A comparative investigation of seven indirect attitude measures. *Behav Res Methods*. 2014;46(3):668-688. doi:10.3758/s13428-013-0410-6

24. Charlesworth TES, Banaji MR. Patterns of Implicit and Explicit Attitudes: IV. Change and Stability From 2007 to 2020. *Psychol Sci*. 2022;33(9):1347-1371. doi:10.1177/09567976221084257

25. Maina IW, Belton TD, Ginzberg S, Singh A, Johnson TJ. A decade of studying implicit racial/ethnic bias in healthcare providers using the implicit association test. *Soc Sci Med*. 2018;199:219-229. doi:10.1016/j.socscimed.2017.05.009

26. Greenwald AG, Nosek BA, Banaji MR. Understanding and using the Implicit Association Test: I. An improved scoring algorithm. *J Pers Soc Psychol*. 2003;85(2):197-216. doi:10.1037/0022-3514.85.2.197

27. Mulchan SS, Miller M, Theriault CB, Zempsky WT, Hirsh A. A Systematic Approach to Developing Virtual Patient Vignettes for Pediatric Health Equity Research. *Health Equity*. 2022;6(1):862-872. doi:10.1089/heq.2022.0108

28. Hirsh AT, George SZ, Bialosky JE, Robinson ME. Fear of Pain, Pain Catastrophizing, and Acute Pain Perception: Relative Prediction and Timing of Assessment. *Journal of Pain*. 2008;9(9):806-812. doi:10.1016/j.jpain.2008.03.012

29. Hirsh AT, George SZ, Robinson ME. Pain assessment and treatment disparities: A virtual human technology investigation. *Pain*. 2009;143(1-2):106-113. doi:10.1016/j.pain.2009.02.005

30. Wandner, Stutts, L. A., Alqudah, A. F., et al. Virtual human technology: patient demographics and healthcare training factors in pain observation and treatment recommendations. *J Pain Res*. Published online December 2010:241. doi:10.2147/jpr.s14708

31. Christison GW, Haviland MG, Riggs ML. *The Medical Condition Regard Scale: Measuring Reactions to Diagnoses*.

32. Devine PG, Forscher PS, Austin AJ, Cox WTL. Long-term reduction in implicit race bias: A prejudice habit-breaking intervention. *J Exp Soc Psychol*. 2012;48(6):1267-1278. doi:10.1016/j.jesp.2012.06.003

33. Haar M. RANDOM.ORG: True Random Number Service.

34. Braun V, Clarke V. Thematic analysis. In: *APA Handbook of Research Methods in Psychology, Vol 2: Research Designs: Quantitative, Qualitative, Neuropsychological, and Biological.* American Psychological Association; 2012:57-71. doi:10.1037/13620-004

35. Gordon Allport. *The Nature of Prejudice.* Addison-Wesley; 1954.

36. Darnall BD, Burns JW, Hong J, et al. Empowered Relief, cognitive behavioral therapy, and health education for people with chronic pain: a comparison of outcomes at 6-month Follow-up for a randomized controlled trial. *Pain Rep*. 2024;9(1):e1116. doi:10.1097/PR9.0000000000001116

37. Tanabe P. It’s Time to Provide Evidence-Based Care to Individuals with Sickle Cell Disease: A Call to Action. *J Emerg Nurs*. 2021;47(5):684-688. doi:10.1016/j.jen.2021.06.002

38. Glassberg JA. Improving Emergency Department-Based Care of Sickle Cell Pain. *Hematology*. 2017;2017(1):412-417. doi:10.1182/asheducation-2017.1.412

39. Zempsky WT, Loiselle KA, McKay K, Lee BH, Hagstrom JN, Schechter NL. Do Children with Sickle Cell Disease Receive Disparate Care for Pain in the Emergency Department? *J Emerg Med*. 2010;39(5):691-695. doi:10.1016/j.jemermed.2009.06.003

40. Smeltzer MP, Howell KE, Treadwell M, et al. Identifying barriers to evidence-based care for sickle cell disease: results from the Sickle Cell Disease Implementation Consortium cross-sectional survey of healthcare providers in the USA. *BMJ Open*. 2021;11(11):e050880. doi:10.1136/bmjopen-2021-050880

41. Forscher PS, Lai CK, Axt JR, et al. A meta-analysis of procedures to change implicit measures. *J Pers Soc Psychol*. 2019;117(3):522-559. doi:10.1037/pspa0000160

42. Wang CJ, Kavanagh PL, Little AA, Holliman JB, Sprinz PG. Quality-of-Care Indicators for Children With Sickle Cell Disease. *Pediatrics*. 2011;128(3):484-493. doi:10.1542/peds.2010-1791

43. Owoo F, Tadros E. The Lived Experiences of Caregivers of Children with Sickle Cell Disease: A Phenomenological Study. *Am J Fam Ther*. 2022;50(5):510-532. doi:10.1080/01926187.2021.1941420

44. Andreychik MR, Gill MJ. Do negative implicit associations indicate negative attitudes? Social explanations moderate whether ostensible “negative” associations are prejudice-based or empathy-based. *J Exp Soc Psychol*. 2012;48(5):1082-1093. doi:10.1016/j.jesp.2012.05.006
